# Antibacterial activity of apramycin at acidic pH warrants wide therapeutic window in the treatment of complicated urinary tract infections and acute pyelonephritis

**DOI:** 10.1016/j.ebiom.2021.103652

**Published:** 2021-11-02

**Authors:** Katja Becker, Sha Cao, Anna Nilsson, Maria Erlandsson, Sven-Kevin Hotop, Janis Kuka, Jon Hansen, Klara Haldimann, Solveiga Grinberga, Talia Berruga-Fernández, Douglas L. Huseby, Reza Shariatgorji, Evelina Lindmark, Björn Platzack, Erik C. Böttger, David Crich, Lena E. Friberg, Carina Vingsbo Lundberg, Diarmaid Hughes, Mark Brönstrup, Per E. Andrén, Edgars Liepinsh, Sven N. Hobbie

**Affiliations:** a Institute of Medical Microbiology, University of Zurich, Gloriastrasse 30, CH-8006 Zurich, Switzerland; b Department of Medical Biochemistry and Microbiology, Uppsala University, Box 582, 751 23 Uppsala, Sweden; c Department of Pharmaceutical Biosciences, Uppsala University, Box 591, 751 24 Uppsala, Sweden; d Science for Life Laboratory, Uppsala University, Box 591, 751 24 Uppsala, Sweden; e RISE Research Institutes of Sweden, Forskargatan 20G, 151 36 Södertälje, Sweden; f Helmholtz Centre for Infection Research, Inhoffenstrasse 7, D-38124 Braunschweig, Germany; g Latvian Institute of Organic Synthesis, Aizkraukles 21, LV-1006 Riga, Latvia; h Statens Serum Institute, Artillerivej 5, DK-2300 Copenhagen, Denmark; i Department of Pharmaceutical and Biomedical Sciences, University of Georgia, 250 W. Green Street, Athens, GA 30602, USA; j Department of Pharmacy, Uppsala University, Box 580, 751 23 Uppsala, Sweden

**Keywords:** Anti-bacterial agents, proton-motive force, delta pH, permeability, drug uptake, urinary tract, efficacy, nephrotoxicity

## Abstract

**Background:**

The clinical-stage drug candidate EBL-1003 (apramycin) represents a distinct new subclass of aminoglycoside antibiotics for the treatment of drug-resistant infections. It has demonstrated best-in-class coverage of resistant isolates, and preclinical efficacy in lung infection models. However, preclinical evidence for its utility in other disease indications has yet to be provided. Here we studied the therapeutic potential of EBL-1003 in the treatment of complicated urinary tract infection and acute pyelonephritis (cUTI/AP).

**Methods:**

A combination of data-base mining, antimicrobial susceptibility testing, time-kill experiments, and four murine infection models was used in a comprehensive assessment of the microbiological coverage and efficacy of EBL-1003 against Gram-negative uropathogens. The pharmacokinetics and renal toxicology of EBL-1003 in rats was studied to assess the therapeutic window of EBL-1003 in the treatment of cUTI/AP.

**Findings:**

EBL-1003 demonstrated broad-spectrum activity and rapid multi-log CFU reduction against a phenotypic variety of bacterial uropathogens including aminoglycoside-resistant clinical isolates. The basicity of amines in the apramycin molecule suggested a higher increase in positive charge at urinary pH when compared to gentamicin or amikacin, resulting in sustained drug uptake and bactericidal activity, and consequently in potent efficacy in mouse infection models. Renal pharmacokinetics, biomarkers for toxicity, and kidney histopathology in adult rats all indicated a significantly lower nephrotoxicity of EBL-1003 than of gentamicin.

**Interpretation:**

This study provides preclinical proof-of-concept for the efficacy of EBL-1003 in cUTI/AP. Similar efficacy but lower nephrotoxicity of EBL-1003 in comparison to gentamicin may thus translate into a higher safety margin and a wider therapeutic window in the treatment of cUTI/API.

**Funding:**

A full list of funding bodies that contributed to this study can be found in the Acknowledgements section.

**Research in context**

**Evidence before this study**

Renal drug clearance results in high transient drug concentrations in the kidney and in urine, which is valuable in attaining therapeutic exposure in the kidney and urinary tract, but may as well translate into an increased risk of nephrotoxicity. Aminoglycoside antibiotics are mainly excreted by glomerular filtration, resulting in very high transient drug exposures of the urinary tract including kidneys, and have therefore been successfully used as antibacterial therapeutics in complicated urinary tract infections and acute pyelonephritis (cUTI/AP). High exposure to aminoglycosides, however, has also been associated with a risk of nephrotoxic adverse effects, in particular in cases of high trough levels and renal drug accumulation over time. The clinical utility of an antibiotic used in the treatment of cUTI/AP is therefore inevitably determined by its therapeutic window, the safety margin between highly efficacious and toxic drug exposures.

The aminoglycoside apramycin, currently in clinical development for the treatment of Gram-negative systemic infections, has demonstrated superior in-vitro activity against highly drug-resistant Gram-negative bacterial pathogens, higher efficacy in preclinical pneumonia models, and lower cochlear toxicity when compared to other aminoglycoside antibiotics. However, its potential efficacy in the treatment of urinary tract and kidney infections, and its potential nephrotoxicity, had not been studied.

**Added value of this study**

This study provides preclinical evidence for the therapeutic potential of apramycin in the treatment of cUTI/AP. Comparison to current standard-of-care aminoglycosides not only suggests better coverage of drug-resistant bacterial pathogens, but also a more robust therapeutic window of higher tolerance towards lower pH values, as commonly encountered in urine but also other host microenvironments of infection.

**Implications of all the available evidence**

The collective evidence implies that antimicrobial susceptibility testing performed at pH 7.4 may not always be fully predictive of the antibacterial potency at the site of infection, that changes in pH may alter the ability of a drug to penetrate bacterial cell walls, and that the specific host environment needs to be considered when extrapolating from laboratory testing results to therapeutic potency in selecting the most appropriate treatment. Conversely, a drug molecule's physicochemical properties in a given environment and their impact on the therapeutic window of that drug may provide for additional clues in the rational design and medicinal chemistry of novel therapeutics.

Alt-text: Unlabelled box

## 1 Introduction

Urinary tract infections (UTI) are reported with a prevalence of more than 10% for women in the USA and are estimated to affect 150 million people worldwide each year [1,2]. As such UTI is recognized as one of the most common bacterial infections encountered by physicians [3,4]). Complicated UTI (cUTI) is defined by the presence of at least one of a number of complicating factors such as pregnancy, diabetes, functional or anatomic abnormalities, immune-compromised conditions, or a drug-resistant infection that withstands standard antibiotic treatment [5,6]. The majority of uropathogens are Gram-negative bacteria of the order *Enterobacterales* with *Escherichia coli* estimated to account for more than 80% of these [4,6]. *Pseudomonas aeruginosa* represents another problematic and often multidrug-resistant (MDR) uropathogen. Multiple scientific reports point to an overall increase in drug-resistant cUTI in recent years with extended-spectrum β-lactamase (ESBL)-positive *Enterobacterales* now commonly encountered [5], [6], [7]. In the SENTRY surveillance program (2007-2009), resistance to carbapenems occurred at a rate of 1.8-2.4% in uropathogenic isolates, a number that is widely assumed to have risen since and is expected to further increase, thus limiting antibacterial treatment options in the future [6,8].

Treatment guidelines for cUTI and pyelonephritis caused by ESBL- or carbapenemase-producing *Enterobacterales* and *P. aeruginosa* demand parenteral therapy with broad-spectrum antibiotics typically including a β-lactam/β-lactamase-inhibitor combination, plus either a fluoroquinolone or an aminoglycoside [6,9]. More recently, however, fluoroquinolones are increasingly avoided for their potential risk of long-term neurotoxic adverse effects [10]. Aminoglycosides, colistin, and tigecycline are alternatives recommended as antipseudomonal drugs in MDR infection and in case of limited treatment options for highly drug-resistant *Enterobacterales* infections. The standard-of-care aminoglycosides in the treatment of cUTI are gentamicin, tobramycin and amikacin with the choice varying as a function of local resistance patterns [5].

Renal clearance of aminoglycoside antibiotics results in very high transient drug concentrations in urine. Single-dose parenteral aminoglycoside as UTI monotherapy has proven a highly effective treatment in a systematic review of 13 studies representing 13 804 patients [11]. However, the antibacterial potency of gentamicin and other antibiotics may at the same time be compromised by the slightly acidic pH typically found in urine [12], [13], [14]. *E. coli* UTI infections have been shown to decrease the naturally acidic urinary pH further to 5.5-6.5 in both adult and paediatric patients [15,16].

Bacterial resistance to aminoglycoside antibiotics is conferred by a variety of mechanisms including the expression of aminoglycoside-modifying enzymes (AME), which inactivate the drug by catalysing alteration of their chemical structures, thus lowering their affinity for the bacterial ribosome [17,18]. Methylation of 16S-ribosomal RNA nucleotide G1405 by ribosome methyltransferases (RMT) results in high-level resistance to all aminoglycoside antibiotics of clinical relevance including the most recent addition plazomicin, and has therefore been termed “pan-aminoglycoside resistance” [9,[17], [18], [19], [20]].

The quest for an improved aminoglycoside antibiotic has identified apramycin as an interesting candidate that naturally overcomes a large majority of all aminoglycoside resistance mechanisms. Indeed, apramycin has been proposed as a next-generation aminoglycoside and is currently undergoing clinical trials as a drug candidate for systemic Gram-negative infections, an effort supported by the European Gram-negative Antibacterial Engine (ENABLE) which is funded by the Innovative Medicines Initiative (IMI) [21]. Apramycin differs from other aminoglycosides by its chemical structure that contains a unique octadiose core, and enables it to evade all circulating RMT-mediated resistance and almost all AMEs. Consequently, the crystalline free base of apramycin under development, designated EBL-1003, has shown promising *in-vitro* and *in-vivo* activity against a broad spectrum of aminoglycoside- and carbapenem-resistant (CR) *A. baumannii, Enterobacterales*, and other critical pathogens [18,[22], [23], [24], [25], [26], [27], [28]]. In the case of *A. baumannii,* EBL-1003 has shown encouraging efficacy in mouse thigh and lung infection models [24,26]. However, additional reports on the preclinical evidence for other sites of infection and other bacterial pathogens has thus far been scarce.

In the present study, we explored the preclinical profile of EBL-1003 against uropathogenic clinical isolates and its therapeutic potential in four mouse UTI infection models. Time-kill analysis was applied to further explore the pH-dependent potency of EBL-1003. Drug concentrations in urine, accumulation in kidneys over repeat dosing, and nephrotoxic effects were studied in mouse and rat models to assess the therapeutic window of EBL-1003 in comparison to that of gentamicin.

## 2 Methods

### 2.1 NDARO database analysis

A genotypic assessment of the antimicrobial susceptibilities of all uropathogenic bacterial isolates deposited in the NCBI National Database of Antibiotic Resistant Organisms (NDARO) was conducted as previously described for other subsets of isolates [26,29]. Resistance gene annotations were downloaded on 16^th^ August 2021, covering clinical isolates from 2012 to 2021, and comprising 12 956 uropathogenic Gram-negative clinical isolates as identified by the following filters. Host: human, homo sapiens, patient; isolation type: clinical; isolation source: urine, urethra, patient with UTI. This filter setting returned a total of 13689 Gram-negative clinical isolates including an unusual high number of 733 *Salmonella enterica*, which are more likely fecal contaminants than true uropathogens. The *S. enterica* isolates have therefore not been included in the subsequent downstream analysis of uropathogens. Aminoglycoside- and carbapenem-resistant genotypes were identified by screening the remaining 12956 gene annotations for any of the relevant resistance genes listed in the NCBI Pathogen Detection Reference Gene Catalogue, as summarized in **Table S2**. A bioinformatic tool has been programmed to accelerate the analysis of the data sets and the code has been deposited in GitLab (https://gitlab.com) for public access.

### 2.2 Antimicrobial susceptibility testing

Minimal inhibitory concentrations (MIC) and time-kill kinetics were determined according to CLSI standards and as previously described [18,26]. Contemporary clinical isolates of bacterial uropathogens with a non-susceptibility to at least one standard-of-care antibiotic were collected between 2019 and 2020 at the University of Zurich's Institute of Medical Microbiology (*n* = 57). MICs and time-kill kinetics were also determined for additional bacterial strains used either as a quality control or in the animal infection studies, listed in **Table S3**. Susceptibility testing and time-kill experiments over a range of defined pH values followed the same protocols using cation-adjusted Müller-Hinton broth (CAMHB) with pH adjusted and buffered at pH 7.4 with 20 mM MOPS, and pH 6 and pH 5 each with 100 mM MES. Buffer concentrations were selected by titration of the minimal tonicity that provided stable pH during bacterial growth for 24 hours (data not shown).

### 2.3 Cellular uptake studies

Bacteria were grown overnight in pH-adjusted and buffered CAMHB at pH 5.0, 6.0 and 7.4 as described above. The cells were suspended in media to an OD_600_ of 0.1 and grown to an OD_600_ of 0.8. After bacterial growth the pH values of the cultures were again measured and determined as pH5.72, pH6.49, and pH7.31, respectively, at the time the antibiotics were added to the cultures. After 10 minutes of incubation at a final antibiotic concentration of 100 µM (*n* = 3 biological replicates for each drug and each pH), subcellular fractionation was performed as previously described by Prochnow *et al.* [30]. In brief, the periplasm was harvested after a cold osmotic shock. Subsequently, cytoplasm and membrane fractions were separated via high-speed centrifugation. Each of the three fractions was then treated as followed: prior to LC analysis, a protein precipitation step was applied using an ice-cold H_2_O/ACN/MeOH (40/30/30%) mixture, followed by a low-speed centrifugation in a 96-well microtitre plate. The supernatant was dried completely followed by resuspension in 40 µl of MS-buffer (95% H_2_O/5%ACN) containing kanamycin as an internal standard. LC/MS/MS-analysis was carried out using an Agilent Infinity II 1290 HPLC (Agilent Technologies, Santa Clara, CA, USA) coupled to an AB Sciex QTrap 6500 triple quadrupole mass spectrometer (AB Sciex Germany GmbH, Darmstadt, Germany). Following an injection of 5 µl of sample, the compounds were separated using a Shodex HILICpak VC-50 2D column equipped with a VC-50G 2A pre-column (both: Showa Denko, K.K., Japan) at a constant flowrate of 0.3 ml/min using a linear gradient of solvents A (ACN, 0.1% HCOOH) and B (H_2_O, 1.5% NH_3_). The linear gradient started at 70% solvent B and reached 90% B at 4.5 min. The compounds were ionized in the positive mode using electrospray ionization. The amount of compound was determined using standard curves for quantification, which were obtained by peak area integration of specific Q3 fragments generated by collision of Q1-filtered molecular masses for each compound in the respective matrix (m/z: apramycin = 540/217, 540/378; gentamicin = 450/322, 450/160, 464/322, 464/160, 478/157, 478/322; amikacin = 586/163, 586/425; kanamycin = 484/163, 484/324).

### 2.4 Mouse efficacy studies

The mouse urinary tract infection model with *E. coli* strain J96 (ATCC 7000336) isolated from a human pyelonephritis patient was subcontracted to Pharmacology Discovery Services (PDS) Taiwan. The efficacy of pH-adjusted aqueous solutions of crystalline apramycin free base (EBL-1003) versus gentamicin sulphate (Sigma Cat# G-3632) was assessed in female C3H/HeJ mice infected by transurethral injection of 9.13 × 10^8^ CFU. Starting six days prior to infection, diuresis was induced by administering 5% glucose in drinking water. Starting 3 days (96 hours) post infection, EBL-1003 or gentamicin were administered subcutaneously twice daily (BID; q12h) for three consecutive days. One control group of mice (*n* = 5) was sacrificed at the start of treatment to determine baseline bacterial counts. Mice from all other dosing groups (*n* = 5 per group) including the vehicle control were sacrificed 7 days (168 h) after infection and bladder and kidney tissue was aseptically removed for bacterial enumeration.

### 2.5 Mouse pharmacokinetic studies

The plasma pharmacokinetics of apramycin in mice has been studied previously [31]. To determine the drug concentration in mouse urine, female NMRI mice (*n* = 4 mice per dose group) received single subcutaneous doses of 0.8, 3.2, or 10 mg/kg of apramycin (Huvepharma, BM8900001, diluted in physiologic saline). Urine samples were collected at 1, 2, 3, 4, 6, and 24 h post administration. Drug concentrations in urine were determined by liquid chromatography tandem mass-spectrometry (LC-MS/MS). In short, protein precipitation was achieved by adding 20 µL of 20% TCA and 470 µL of aqueous solution of 0.01% HFBA and 0.01% propionic acid to 10 µL of urine. Samples were centrifuged for 10 min at 11000 × *g* and 1 µL of each supernatant was injected into the LC-MS/MS system (Waters XEVO-TQ-S).

### 2.6 Mass Spectrometry Imaging

Kidney tissue samples were collected from control animals (CD-1 mice, Envigo) and animals sacrificed at 1 h, 2 h, and 4 h post administration of 100 mg/kg EBL-1003 and immediately frozen and stored at -80°C. Kidney samples were cut in sagittal sections using a cryostat-microtome (Leica CM1900; Leica Microsystems, Wetzlar, Germany) at a thickness of 12 μm, thaw-mounted onto conductive indium tin oxide (ITO) glass slides (Bruker Daltonics, Bremen, Germany), and further stored at -80°C until matrix-assisted laser desorption ionization (MALDI)-mass spectrometry imaging (MSI) analysis. On the day of analysis, tissue samples were transported on dry ice and then brought to room temperature in a vacuum desiccator. After drying for 30 min the slide was scanned on a flatbed scanner (Epson perfection V500). Both the internal standard solution (2.5 mg of kanamycin in 6 ml 50% MeOH) and the MALDI matrix 2,5-dihydroxybenzoic acid (DHB) (35 mg/ml in 50% acetonitrile, 0.2% trifluoroacetic acid) was applied to the slide in 6 passes using the TM-sprayer (HTX Imaging, Chapel Hill, NC, USA) with the following parameters: temperature 95°C, flow rate 70 μL/min, nozzle velocity 1100 mm/min, track spacing 2.0 mm, and N_2_ pressure was set to 6 psi. MALDI-MSI experiments were performed on a MALDI Fourier-transform ion cyclotron resonance (FTICR) mass spectrometer (solariX 7T-2ω, Bruker Daltonics). Prior to analysis, the method was calibrated using red phosphorus. Online calibration was performed using a DHB matrix peak at *m*/*z* 273.0394. Data was collected in positive ionization mode in the mass range of m/z 150–1000 and Q1 mass was set to *m*/*z* 250. Transient size 2M and 2ω was used resulting in a mass resolution of 260,000 at *m*/*z* 369. Time-of-flight value was set to 0.600 ms and transfer optics frequency was 4 MHz. The small laser size setting was used and spectra were collected from 100 shots per pixel. Data were collected at 100 µm and 30 µm lateral resolution. Fleximaging 5.0 (Bruker Daltonics) was used for data visualization and image extraction. Following MSI analysis the MALDI matrix was removed by submerging the slide in 95% ethanol for 30 s and then the tissue sections were stained by haematoxylin and eosin. The H&E images were imported to FlexImaging and overlaid with the MSI data acquired at 30 µm lateral resolution.

### 2.7 Rat pharmacokinetic and toxicity studies

Drug concentrations in rat kidneys were analysed in male 16-week-old Sprague Dawley rats after subcutaneous administrations of EBL-1003 at doses of 25, 50 or 100 mg/kg or gentamicin at a dose of 3 mg/kg (KRKA, Slovenia, external batch number A62904). One cohort of animals (*n* = 3 per dose) was dosed once daily with EBL-1003 or gentamicin for five consecutive days (receiving 5 doses) and another group of animals (*n* = 5 per dose) was dosed once daily for 8 consecutive days (receiving 8 doses). Animals were humanely sacrificed two hours after injection of the last dose and drug concentrations in kidney tissue were measured by LC-MS/MS analysis.

For nephrotoxic assessment of apramycin relative to gentamicin, adult 14-18-week-old male Sprague Dawley rats were dosed once daily for 21 days with 12.5, 25, 50, or 100 mg/kg of subcutaneous apramycin (Biovet, Huvepharma, Bulgaria); or 3, 10, or 30 mg/kg of subcutaneous gentamicin for injections (KRKA) (*n* = 5 animals per dose group). For pharmacokinetic analysis, blood samples were obtained from the 100 mg/kg apramycin dosing group at selected time points (15, 30, 60, 120, 240 min post injection) on day 1, day 7 and day 14 of treatment. Apramycin concentrations in plasma and kidney tissue were analysed by LC-MS/MS. Compound accumulation was assessed in kidney tissue after 14 days of treatment. Recovery after the regimen was determined by monitoring of BUN, creatinine in plasma, and KIM-1 secretion in urine up to day 35. Formalin-fixed kidneys were stained with haematoxylin-eosin and histopathology evaluated by light microscope.

### 2.8 Ethics

All aspects of this work, including housing, experimentation, and disposal of animals were performed in general accordance with the Guide for the Care and Use of Laboratory Animals (National Academy Press, Washington, D. C., 2011). The murine efficacy studies were performed in an AAALAC accredited ABSL2 laboratory with animal care and use protocols approved by the Institutional Animal Care and Use Committee (IACUC) at Eurofins Panlabs Taiwan (Fig. 2); by the National Committee of Animal Ethics, Ministry of Environment and Food of Denmark (Fig. S2); or by the IACUC of the University of Texas Medical Branch (Fig. S3). Pharmacokinetic and toxicology studies in mice and rats were performed under international and local laws and policies and were approved by either the Latvian Animal Protection Ethical Committee, Food and Veterinary Service, Riga, Latvia (Fig. 4a, 4c-h, S5, S6, and S7) or by the Swedish national ethical license S7-15 (Fig. 4b).

### 2.9 Statistics

One-way ANOVA followed by Dunnett's comparison test was employed to assess significance of difference between efficacy treatment and control groups. Student's t-test was used in the comparison of drug exposure in infected vs. non-infected mice.

### 2.10 Role of the funding source

The funding sources played no role in the study design, data collection, data analysis, interpretation, writing of the report, and the decision of paper submission.

## 3 Results

### 3.1 Genotypic antibiotic resistance of uropathogenic isolates deposited in the National Database of Antibiotic-Resistant Organisms (NDARO)

First, we identified the number and aetiology of clinical isolates of uropathogenic origin deposited in the NDARO. We found *Enterobacterales* to be the predominant group of Gram-negative uropathogens, with 5456 (42.1%) *Klebsiella pneumoniae* and 5049 (39.0%) *E. coli* isolates deposited in the NDARO at the time of analysis. Other bacterial species of urinary origin in the NDARO were *Pseudomonas aeruginosa* (*n* = 672, 5.2%), *Enterobacter* spp. (*n* = 488, 3.8%), and other *Enterobacterales* (collectively *n* = 565, 4.4%). The relative prevalence of individual bacterial species deposited in the NDARO as potential uropathogens resembles that of previous cUTI surveillance studies (**Table S1**), apart from an apparent over-representation of *K. pneumonia* in the NDARO, a data base with an intrinsic bias of collecting data on drug-resistance isolates only.

**Fig. 1:**
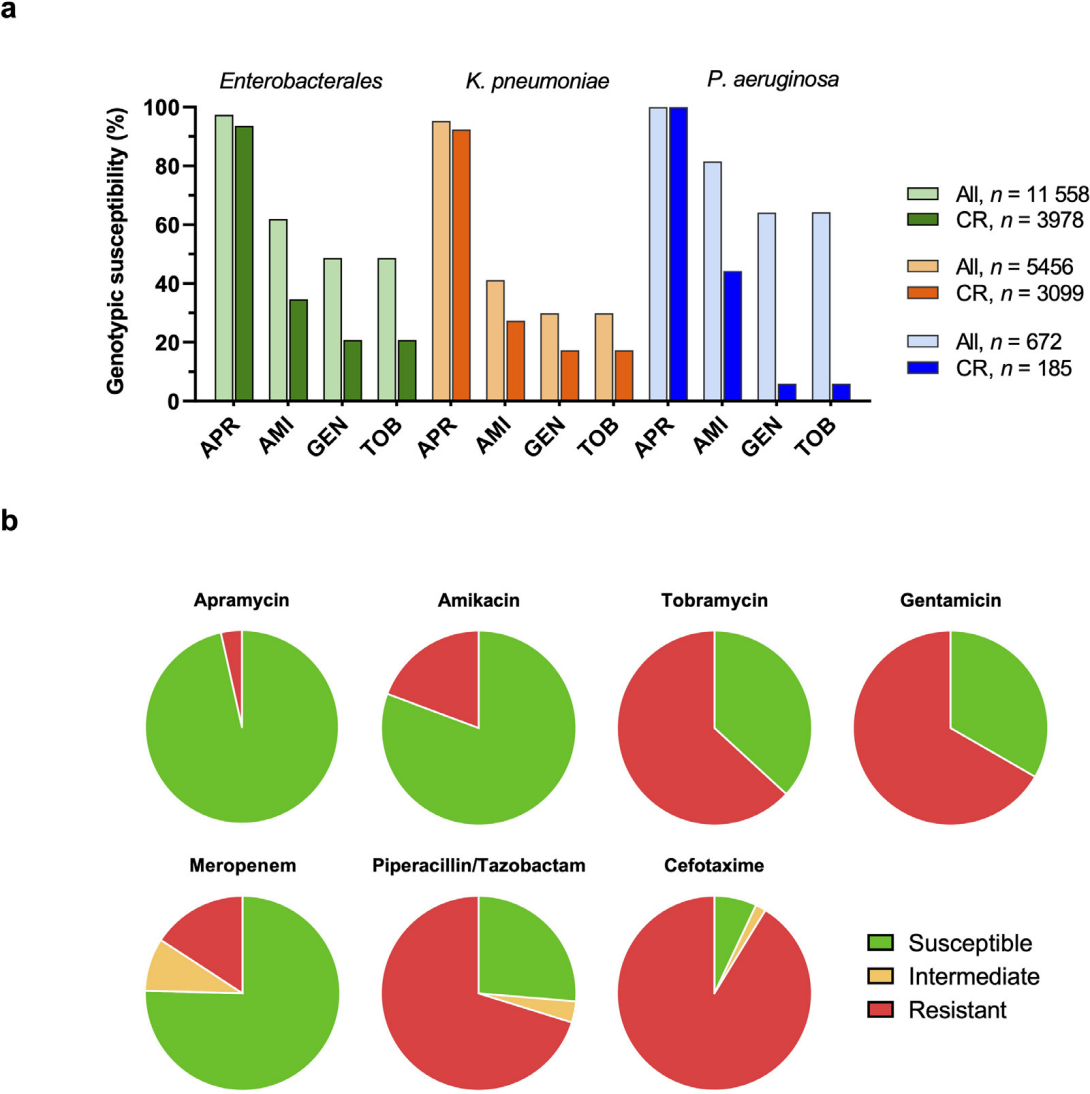
*Genotypic and phenotypic antimicrobial susceptibility of uropathogenic isolates.* (**a**) Genotypic aminoglycoside susceptibility of 11 558 urinary Gram-negative isolates deposited in the National Database of Antibiotic Resistant Organisms (NDARO). Genotypic susceptibility was defined by the absence of resistance gene annotations known to affect the susceptibility to a specific drug. The *Enterobacterales* group comprised *E. coli*/*Shigella, Klebsiella* spp., *Enterobacter* spp., *Citrobacter freundii, Morganella morganii, Serratia marcescens*, and *Providencia alcalifaciens*. Genetic resistance determinants applied in the analysis are listed in Table S2. APR, apramycin; AMI, amikacin; GEN, gentamicin; TOB, tobramycin; CR, carbapenem resistance. (**b**) Phenotypic antimicrobial susceptibility of a panel of 57 contemporary non-susceptible uropathogenic isolates comprising *E. coli* (*n* = 20), *K. pneumoniae* (*n* = 10), *K. oxytoca* (*n* = 5), *E. cloacae* (*n* = 5), *P. mirabilis* (*n* = 8), and *P. aeruginosa* (*n* = 9). EUCAST 2021 interpretative criteria for *Enterobacterales* and *P. aeruginosa* were applied to the minimal inhibitory concentrations (MICs) listed in Table S4. For apramycin, epidemiologic cutoff-values (ECOFFs) of 16 mg/L for *Enterobacterales* and 32 mg/L for *P. aeruginosa* were used as interpretative criteria. MIC distributions are plotted in Fig. S1.

Second, we assessed the genotypic resistance profile of uropathogenic *Enterobacterales* and *P. aeruginosa* isolates deposited in the NDARO based on their annotated resistance genes. The analysis of 12 956 deposited uropathogenic, Gram-negative genomes revealed that genotypic susceptibility to apramycin was generally high among *Enterobacterales* (97.4%) and *P. aeruginosa* (100%) isolates (Fig. 1**a**). In contrast, isolates of all species, and in particular *K. pneumoniae,* showed reduced genotypic susceptibility to gentamicin, tobramycin and amikacin. Carbapenem resistance genes were annotated in 34.4% of uropathogenic *Enterobacterales* overall but occurred with a considerably high prevalence in urinary *K. pneumoniae* isolates (56.8%). A particularly low genotypic susceptibility to standard-of-care drugs was observed for these carbapenem-resistant populations of *Enterobacterales* and *P. aeruginosa* isolates. In contrast, apramycin susceptibility was retained in the vast majority of CR uropathogenic isolates of all species.

### 3.2 *In-vitro* activity of EBL-1003 against uropathogenic isolates

Next, we investigated the *in-vitro* activity of apramycin in comparison to standard-of-care drugs against a panel of contemporary uropathogenic *Enterobacterales* and *P. aeruginosa* collected at the University of Zurich diagnostic laboratories. Broth microdilution assays with 57 isolates indicated a modal MIC of 2 mg/L (*K. pneumoniae*) to 8 mg/L (*P. aeruginosa*) for apramycin depending on species. The resistance phenotypes are summarized in Fig. 1**b**, MIC distributions of apramycin in comparison to amikacin, tobramycin, gentamicin, piperacillin/tazobactam, cefotaxime, and meropenem are plotted in **Fig. S1**, and the individual MICs for each strain are summarized in **Table S4**. Susceptibility to apramycin was generally high including RMTase-positive pan-aminoglycoside resistant *E. coli, K. pneumoniae* and *Proteus mirabilis* isolates.

ESBL and resistance to third-generation cephalosporins (3-GC) was of high prevalence in the tested panel of UTI isolates with a non-susceptibility rate of 94.7% for cefotaxime and 73.7% for piperacillin/tazobactam. Carbapenem resistance occurred in 24.6% of isolates as identified by non-susceptibility to meropenem. Apramycin showed the lowest resistance rate (3.5%) among the tested aminoglycosides in this panel followed by amikacin (19.3%). The bactericidal activity of apramycin was also confirmed in time-kill assays with a drug-susceptible uropathogen, showing 4-8-fold lower potency than gentamicin at neutral pH, a finding that corresponded well to the difference in MICs between apramycin (4 mg/L) and gentamicin (0.5-1 mg/L) (**Fig. S4**).

### 3.3 *In vivo* efficacy of EBL-1003 in murine urinary tract infection models

**Fig. 2:**
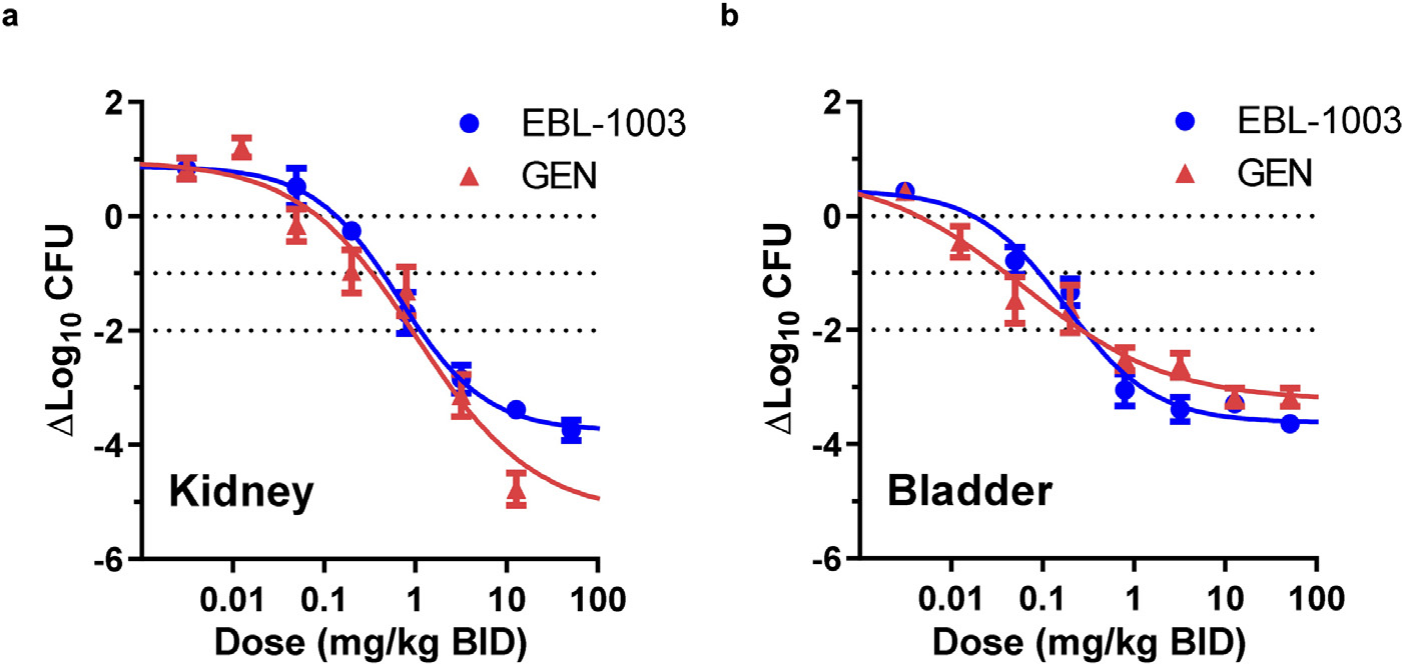
***In-vivo****efficacy of EBL-1003 (apramycin) in comparison to gentamicin in a murine cUTI model infected with the uropathogen E. coli J96.* Female C3H/HeJ mice were infected with 9.13 × 10^8^ CFU/mouse by transurethral injection in the bladder and treated subcutaneously with twice daily doses of EBL-1003 (MIC = 4 μg/mL) or gentamicin (GEN, MIC = 0.5–1 μg/mL) for three days starting 96 h post infection. (**a**) Dose-response multi-log CFU reduction in the kidney. One-way ANOVA and Dunnett's test of CFU reduction relative to start of treatment resulted in *p*< 0.05 for doses ≥0.8 mg/kg BID of EBL-1003 or gentamicin. (**b**) Dose-response multi-log CFU reduction in the bladder. One-way ANOVA and Dunnett's test of CFU reduction relative to start of treatment resulted in *p*< 0.05 for doses ≥0.2 mg/kg BID of EBL-1003 or gentamicin. Data blotted as mean ± SEM CFU reduction in *n* = 5 mice per dose group.

We further assessed the drug-candidate EBL-1003 (apramycin for injection) in comparison to gentamicin in a murine model of cUTI infection with the uropathogenic *E. coli* J96 strain. Here, apramycin induced a higher than tenfold reduction in CFU counts in bladder and kidney at a dose of 0.8 mg/kg, BID, q12h. The highest dose of 51.2 mg/kg apramycin resulted in an almost 4-log_10_ reduction in CFU counts in both organs. This dose in an 18 g mouse is predicted to result in the same drug exposure as a dose of 3.6 mg/kg in a 70 kg human based on allometric scaling principles [31]. The potency of EBL-1003 was comparable to that of gentamicin, which induced a similar log_10_ CFU reduction at dose of 0.8 mg/kg in this UTI infection model (Fig. 2).

The *in vivo* efficacy of EBL-1003 was also confirmed in two additional infection models, one with a pan-aminoglycoside resistant *E. coli rmtB* isolate (strain EN0591), and another with an *E. coli* isolate with an MIC corresponding to the MIC_90_ of 8 mg/L (strain EN0335) (**Fig. S2**). Additionally, apramycin proved efficacious in a diabetic murine model for cUTI infection with the fluoroquinolone-resistant uropathogen *E. coli* M072. Here, the drug-candidate induced a significant reduction of CFU counts in kidney and bladder for doses of 1 mg/kg and higher (**Fig. S3**).

### 3.4 Antibacterial potency at acidic pH

The efficacious potency of EBL-1003 in the murine cUTI model relative to gentamicin was somewhat unexpected considering a 4-8-fold difference in MIC for *E. coli* J96. We therefore decided to further study the susceptibility of *E. coli* J96 *in vitro* at varying culture conditions.

**Fig. 3:**
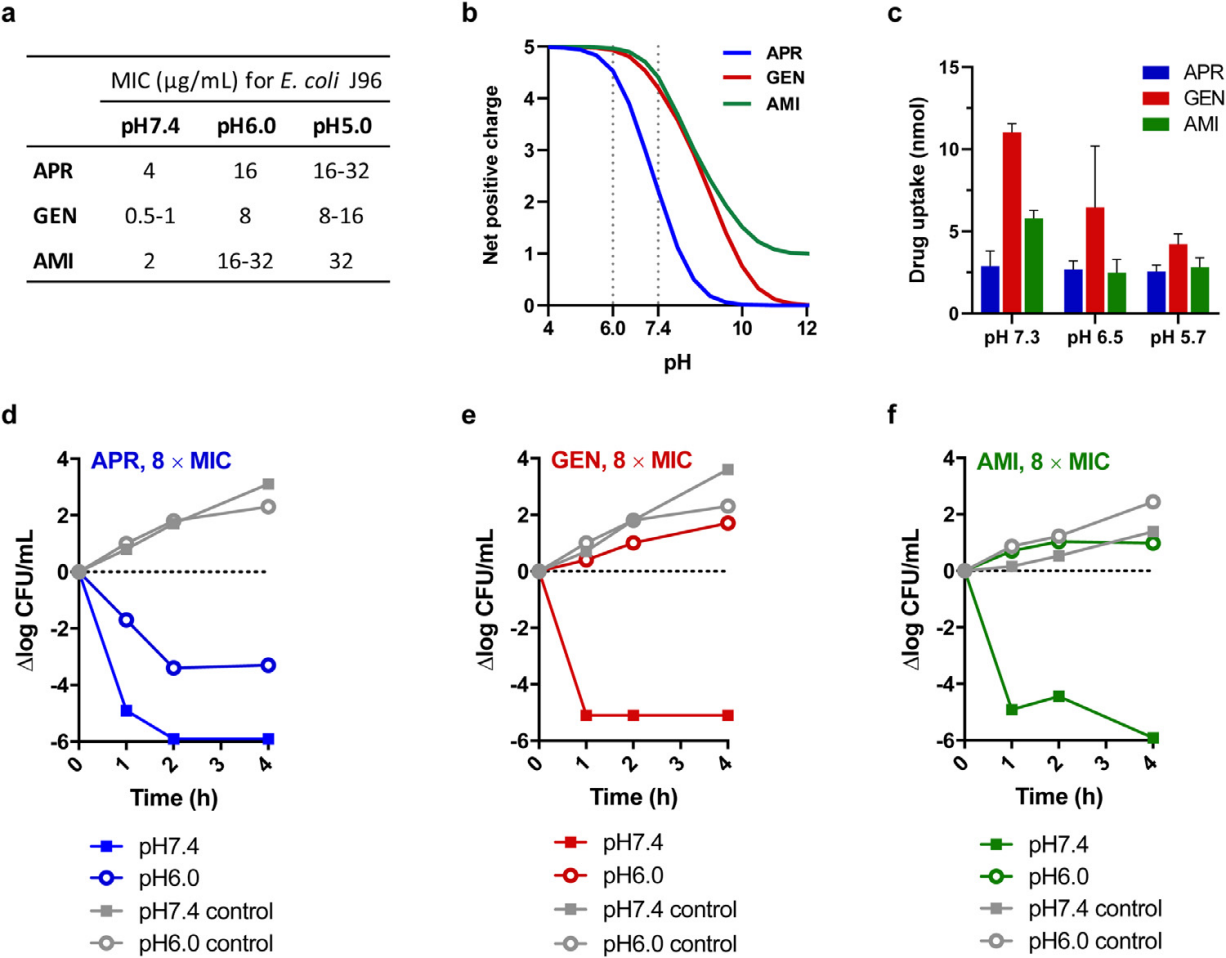
*The effect of pH on the antibacterial activity, positive charge, drug uptake, and time-kill kinetics of apramycin, gentamicin, and amikacin in the uropathogen E. coli J96.***(a**) MICs for *E. coli* J96 at pH7.4, pH6.0, and pH5.0. **(b**) Net positive charge of apramycin, gentamicin, and amikacin when applying the Henderson-Hasselbalch equation to the p*K*_a_ values of individual amines published previously. (**c**) Cytoplasmic uptake of apramycin, gentamicin and amikacin by *E. coli* J96 cells at pH 5.7, pH 6.5, and pH 7.3 quantified by LC/MS/MS following cellular fractionation of 3.9 × 10^9^ cells. Data blotted as mean ± SD of *n* = 4 replicates. (**d-f**) Time-kill kinetics of apramycin (d), gentamicin (e), and amikacin (f) against *E. coli* J96 at a drug concentration of eight times the MIC at neutral pH: 32 μg/mL apramycin, 4 μg/mL gentamicin, and 16 μg/mL amikacin, respectively.

Antimicrobial susceptibility testing of *E. coli* J96 in cation-adjusted Müller-Hinton broth (CAMHB) at neutral pH confirmed an apramycin MIC that was 4- to 8-fold higher than that of gentamicin and about twofold higher than that of amikacin. Interestingly, lowering the pH raised the MIC of all three aminoglycosides, but not to the same extent for each. As a result, the difference in antibacterial potency between the three aminoglycosides appeared to be less pronounced at slightly acidic conditions (Fig. 3**a**). We next repeated antimicrobial susceptibility testing in urine instead of CAMHB and found an even more pronounced increase in MIC with decreasing pH for all drugs (**Table S5**). The improved relative potency of apramycin at acidic pH when compared to gentamicin and amikacin was also observed in time-kill assays with *E. coli* J96 (Fig. 3**d–f** and **Fig. S4**).

We hypothesized that pH-dependent uptake might play a role in improved relative potency of EBL-1003 at urinary pH. To investigate this, drug-uptake into bacterial cells was quantified using a protocol that couples bacterial fractionation to a mass spectrometry-based readout [30]. The major amount of all aminoglycosides was found in the cytoplasm, where the ribosomal target is localized. While lowering the pH from 7.3 to 5.7 resulted in an 11% decrease in cytoplasmic uptake of apramycin, the amounts of gentamicin and amikacin were reduced by 62% and 51%, respectively (Fig. 3**c**).

### 3.5 Pharmacokinetics and nephrotoxicity of EBL-1003 in comparison to gentamicin

**Fig. 4:**
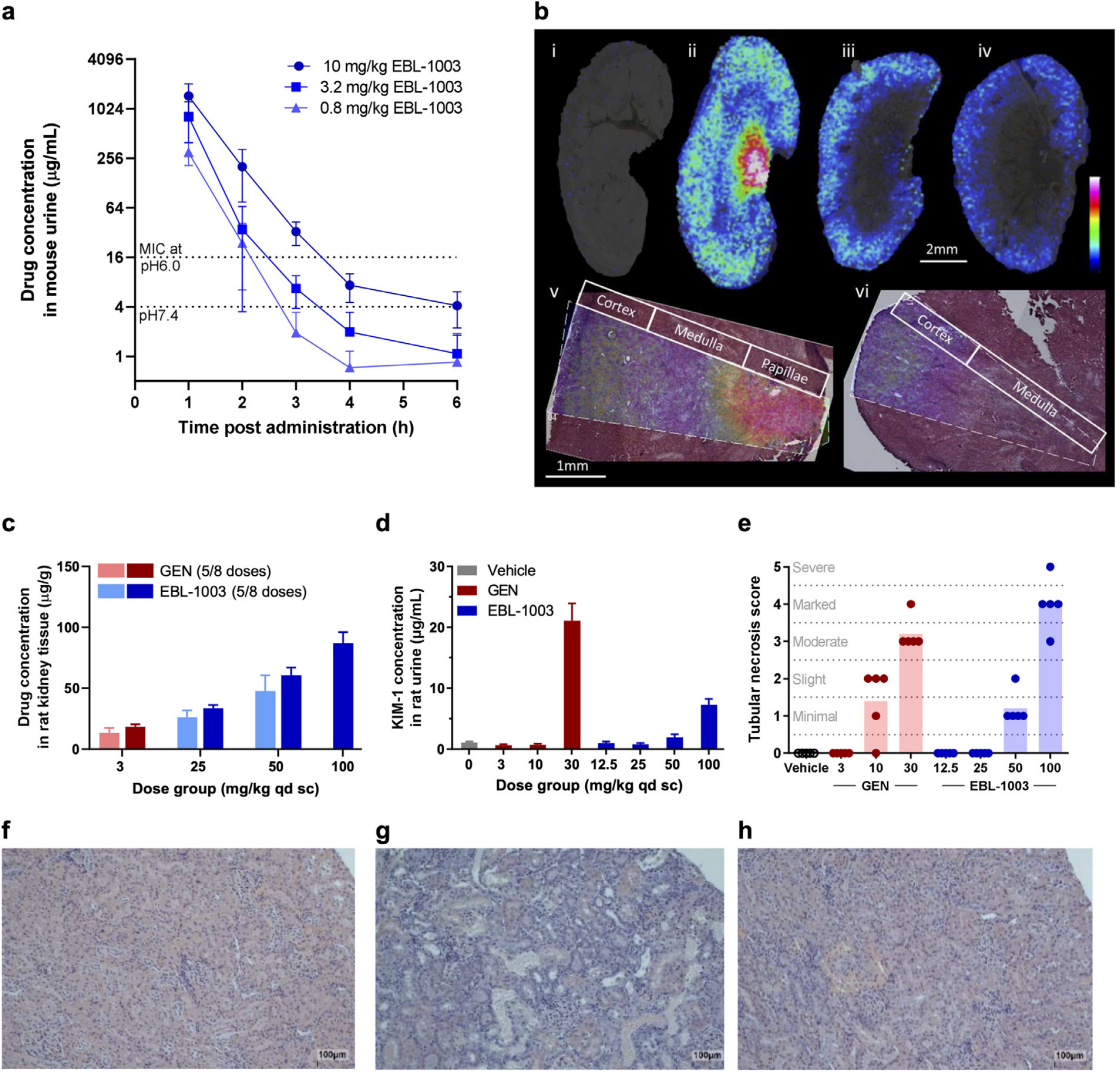
*Drug exposure in urine and kidney and nephrotoxicity of EBL-1003 (apramycin) in comparison to gentamicin. (a)* Concentration of apramycin in mouse urine up to 6 h after administration of EBL-1003. Data blotted as mean ± SD of *n* = 4 animals. (**b**) EBL-1003 distribution in mouse kidney at different time points post administration. i–iv, Ion distribution images of apramycin (*m/z* 540.29) in kidney sections from control (i), 1 h (ii), 2 h (iii), and 4 h (iv) post administration. v-vi, ion distribution of apramycin (*m/z* 540.29) overlaid on the H&E-stained analysed section from a kidney tissue section 1 h post administration (v) and 2 h post administration (vi). Images were acquired at a lateral resolution of 100 µm (i–iv) and 30 µm (v, vi). Data is normalized to internal standard (kanamycin, *m/z* 485.24). Data are shown using a rainbow scale scaled to 0-60% of max intensity. (**c**) Accumulation of apramycin in rat kidneys after repeated dosing of EBL-1003 over 5 to 8 days in comparison to gentamicin (GEN). Tissue concentration was determined 2 h after the final dose. Data blotted as mean ± SD of *n* = 3 rats receiving five doses each, and *n* = 5 rats receiving eight doses each. (**d**) KIM-1 concentration in rat urine after 14 days of repeat dosing. Data blotted as mean ± SD of *n* = 5 replicates. **(e**) Adult rat kidney histopathology scoring after 14 days of repeat dosing of gentamicin or EBL-1003. A score of zero is equivalent to “no finding”; 1, minimal; 2, slight; 3, moderate; 4, marked; 5, severe. (**f–h**) Histopathological cross sections of kidney cortex of adult rats treated with vehicle (**f**), 30 mg/kg gentamicin showing marked tubular necrosis and regeneration (**g**), or 50 mg/kg of EBL-1003 showing minimal tubular necrosis and regeneration (**h**). Tubular necrosis was characterized by cytoplasmic eosinophilia, nuclear pyknosis or karyorrhexis, and sloughing of affected epithelium into tubular lumina with attenuation of the tubular epithelial layer. Tubular regeneration was observed as basophilic lower epithelium with mitotic activity. Scale bar indicates 100 μm.

The promising *in-vitro* and *in-vivo* activity of EBL-1003 motivated us to also explore the main pharmacokinetic characteristics of this compound. The plasma PK of apramycin in mice has previously been described to follow classical aminoglycoside characteristics [31]. Renal clearance resulted in high transient drug concentrations in mouse urine of 302–1458 µg/mL one hour post administration of 0.8-10 mg/kg (Fig. 4**a**) and was thus comparable to gentamicin (**Fig. S5c**). No metabolites of the compound were detected.

MALDI-MS imaging of apramycin in the kidneys of treated mice confirmed the renal clearance, distribution of apramycin across renal tissue, and decreasing apramycin concentrations over time (Fig. 4**b**). High resolution imaging (30 µm) and histologic analysis (HE stain) visualized localization of the drug particularly to the cortex at 2 h after administration when the highest transient concentration in the medulla and papillae had cleared. The concentration of apramycin in renal tissue was dose-dependent and achieved higher concentrations in infected versus non-infected animals (**Fig. S5a**). Repeated twice daily administration resulted in drug accumulation as indicated by higher apramycin concentrations of up to 150 μg/g kidney tissue after six doses (**Fig. S5b**) when compared to just 60 μg/mL after the first dose (**Fig. S5a**), and the accumulation was again found to be more pronounced in infected mice than in uninfected animals.

In 4- and 7-day dosing regimens of EBL-1003 versus gentamicin in the Sprague Dawley (SD) adult rat model, accumulation of apramycin in the kidney tissue appeared to be considerably lower than for gentamicin when set into relation to the administered doses (Fig. 4**c**). The urinary secretion of KIM-1, a sensitive marker for damage in proximal tubular cells [32,33], was found to be the most sensitive of the tested biomarkers for kidney injury (Fig. 4**d**), followed by ß2-microglobulin, clusterin, and albumin (**Fig. S6**). Treatment with EBL-1003 consistently required higher dose levels than gentamicin to trigger comparable nephrotoxic effects. Changes in BUN and creatinine were less pronounced in comparison for both EBL-1003 and gentamicin (data not shown).

Histologic analyses by a trained pathologist showed that a gentamicin dose of 10 mg/kg induced nephrotoxicity scores comparable to those induced by 50 mg/kg of EBL-1003 (Fig. 4e-h). Rats treated with high doses of gentamicin did not survive treatment for more than 5 to 10 days (Fig. S7). High doses of apramycin, however, were tolerated for a full 21-day regimen followed by rapid kidney recovery after the end of treatment (**Fig. S7**).

## 4 Discussion

This study presents first proof-of-concept in animals of the efficacy of drug candidate EBL-1003 in urinary tract infections and a first quantitative assessment of its nephrotoxicity. The efficacious potency of EBL-1003 against the uropathogen *E. coli* J96 was found to be similar to that of gentamicin, whereas its nephrotoxicity in adult rats was found to be significantly lower than that of gentamicin. This suggests a wider therapeutic window for EBL-1003 than for gentamicin in the treatment of urinary tract infections.

An *in silico* database screening indicated a very low prevalence of apramycin resistance genes in *Enterobacterales* including difficult-to-treat CR isolates, which is in agreement with the overall low prevalence of the *aac(3)-IV* resistance gene reported previously [29]. The 3-*N*-acetyltransferase AAC(3)-IV has been shown to be the only clinically relevant resistance mechanism against apramycin, and was recently reported to be less prevalent than all other aminoglycoside-inactivating enzymes including RMTases [29].

Consequently, EBL-1003 (apramycin) demonstrated the highest susceptibility rates in a phenotypic assessment of uropathogenic bacterial isolates. The antibacterial potency of apramycin in broth microdilution assays was confirmed to be lower than that of gentamicin, more closely resembling that of amikacin. It was therefore surprising to find the efficacious potency of EBL-1003 *in vivo* resembling the dose-response curve of gentamicin. We hypothesized that this could be either a pharmacokinetic effect or perhaps an effect of the physiologic difference between cation-adjusted Müller-Hinton broth (CAMHB) and the urethral environment. We have previously reported the pharmacokinetic of EBL-1003 to be similar to that of gentamicin [31]. Antimicrobial susceptibility testing in standard CAMHB (at neutral pH), versus pH-adjusted CAMHB and urine provided a first clue that the antibacterial activity of EBL-1003 is less affected by changes in pH than that of gentamicin or amikacin. It has long been known that the antibacterial activity of aminoglycoside antibiotics is reduced in acidic environments due to reduced drug uptake [12,14]. We found that apramycin follows this trend, too, but that drug uptake and susceptibility are less affected by lower pH than is the case for gentamicin and amikacin.

Early analyses of aminoglycoside uptake mechanisms have shown that the electric potential (Δψ) across the bacterial membrane plays a crucial role in the energy-dependent uptake of gentamicin into the cell, and that an acidic environment lowered the membrane potential, resulting in reduced drug uptake [34,35]. While the mechanism behind the differential effect of pH on EBL-1003 has yet to be elucidated in detail, it is conceivable that the lower degree of protonation of the apramycin molecule in acidic conditions partially compensates for the reduced membrane potential. Previous p*K*_a_ studies indicate that the amino groups of apramycin are generally less basic than those of gentamicin, amikacin, and tobramycin meaning that they will be protonated to a lesser extent at the acidic urine pH [36], [37], [38], [39]. Of particular note are the p*K*_a_s of the two least basic amino groups in apramycin (N-3 and N-4″), which are only 14% protonated at physiologic pH when applying the Henderson-Hasselbalch equation, resulting in a net positive charge of 2.2 for apramycin at pH7.4. In comparison, the net positive charge of gentamicin and amikacin at pH7.4 is about twofold higher (**Fig. S3, Table S6**). Thus, there is a proportionately greater increase in the protonation level of apramycin on going to the more acidic urine at pH6.0, and it is presumably this greater increase in protonation levels that allows apramycin to compensate for a decrease in proton motive force at more acidic conditions.

As we recently reported, the pharmacokinetic parameters of apramycin are in line with expectations from the classical aminoglycoside profile [31]. Rapid clearance almost exclusively by the renal pathway, resulting in very high drug concentrations in urine as is well known for other aminoglycoside antibiotics, is therefore not surprising. Apramycin concentrations appeared to be slightly higher than gentamicin concentrations in urine (at a dose of 10 mg/kg) but this difference alone does not explain the extent of the higher-than-expected *in-vivo* efficacy observed.

We observed a lower level of apramycin accumulation in the kidneys of healthy and infected mice compared to gentamicin and studied the nephrotoxicity profile of both drugs in direct comparison. Histology analysis, KIM-1 secretion as a biomarker for proximal tubular cell (PTC) damage as well as survival studies consistently supported lower nephrotoxicity of apramycin than gentamicin. The uptake of this drug into PTCs was indicated by MS imaging as well, by which apramycin was confirmed to localize to the kidney cortex where PTCs are located.

This study presents an interesting case of how the in-vitro activity of an antibacterial drug candidate may not always be directly predictive of the efficacy in vivo, for reasons that may go beyond the pharmacokinetic profile of a drug candidate. Although we presented a number of observations that are in support of our hypothesis, it is currently based on a limited data set for only a single bacterial pathogen, the UTI/pyelonephritis model organisms *E. coli* J96. More research will be required to probe whether the observations presented here are transferable to other uropathogens, other antibacterial therapeutics, and perhaps other sites of infection with altered pH. Other sites of infection with sub-physiologic pH may include metabolic or drug-induced acidosis in sepsis patients [40,41], hypercapnia-driven respiratory acidosis [42], lysosomal compartments, and the airway surface liquid in Cystic Fibrosis patients, which has been suggested to contribute to increased susceptibility to lung infections, although the debate has remained controversial [43].

The drug-candidate EBL-1003 is currently in Phase I clinical development. Based on the promising efficacy observed in four UTI animal models and its low nephrotoxicity, EBL-1003 holds promise for a novel best-in-class aminoglycoside therapeutic. EBL-1003 may be of particular therapeutic value in complicated and aminoglycoside-resistant infections, and for those patients that benefit from a higher safety margin and therefore a wider therapeutic window.

## Contributors

KB, EL, and SNH conceptualized the studies; KB, SC, AN, ME, SKH, CVL, DH, MB, PEA, EL, and SNH designed the studies; KB, SC, AN, ME, SKH, JK, JH, KH, SG, TBF, DLH, RS, and EL conducted the experiments, collected, and analysed the data; KB, BP, ECB, DC, LEF, CVL, DH, MB, PEA, EL, and SNH were responsible for data analysis and interpretation; KB, ECB, DC, LEF, CVL, DH, MB, PEA, EL, and SNH contributed to manuscript writing. All authors reviewed and approved the manuscript prior to submission.

## Data Sharing

All relevant data have been presented in the manuscript or in the supplementary materials. Source data can be made available upon request to the corresponding author.

## Declaration of Competing Interest

Authors ECB, DC, and SNH are co-founders of and shareholders in Juvabis AG. All other authors declare no conflict of interest.

## Appendix Supplementary materials

Image, application 1

Image, application 2

Image, application 3
