# Supplementary material for: Antibacterial activity of apramycin at acidic pH warrants wide therapeutic window in the treatment of complicated urinary tract infections and acute pyelonephritis
Source: eBioMedicine. 2021 Nov 2;73:103652. doi: 10.1016/j.ebiom.2021.103652 (PMC8577399; doi:10.1016/j.ebiom.2021.103652)
Supplement: Supplementary file 1 [file mmc1.pdf]

The ENABLE consortium has been introduced and described in a *Nature Reviews Drug Discover* article in 2021. Supplementary Table 2 provides a list of consortium members and their affiliations.

Nat Rev Drug Discov. 2021 Jun;20(6):407-408. doi: 10.1038/d41573-021-00074-y.

ENABLE: an engine for European antibacterial drug discovery and development

Marie Olliver, Laura Griestop, Diarmaid Hughes, Anna Karin Belfrage, Johan Gising, Pawel Baranczewski, Carina Vingsbo Lundberg, Anders Karlén
