# Supplementary material for: Antibacterial activity of apramycin at acidic pH warrants wide therapeutic window in the treatment of complicated urinary tract infections and acute pyelonephritis
Source: eBioMedicine. 2021 Nov 2;73:103652. doi: 10.1016/j.ebiom.2021.103652 (PMC8577399; doi:10.1016/j.ebiom.2021.103652)
Supplement: Supplementary file 2 [file mmc2.pdf]

Supplementary material to doi:10.1016/j.ebiom.2021.103652

Antibacterial activity of apramycin at acidic pH warrants wide therapeutic window in the treatment of complicated urinary tract infections and acute pyelonephritis

Katja Becker [a], Sha Cao [b], Anna Nilsson [c, d], Maria Erlandsson [e], Sven-Kevin Hotop [f], Janis Kuka [g], Jon Hansen [h], Klara Haldimann [a], Solveiga Grinberga [g], Talia Berruga-Fernández [b], Douglas L. Huseby [b], Reza Shariatgorji [c, d], Evelina Lindmark [e], Björn Platzack [e], Erik C. Böttger [a], David Crich [i], Lena E. Friberg [j], Carina Vingsbo Lundberg [h], Diarmaid Hughes [b], Mark Brönstrup [f], Per E. Andrén [c, d], Edgars Liepinsh [g], Sven N. Hobbie [a, *]

[a] University of Zurich, Institute of Medical Microbiology, Zurich, Switzerland
[b] Uppsala University, Department of Medical Biochemistry and Microbiology, Uppsala, Sweden
[c] Uppsala University, Department of Pharmaceutical Biosciences, Uppsala, Sweden
[d] Uppsala University, Science for Life Laboratory, Uppsala, Sweden
[e] RISE Research Institutes of Sweden, Södertälje, Sweden
[f] Helmholtz Centre for Infection Research, Braunschweig, Germany
[g] Latvian Institute of Organic Synthesis, Riga, Latvia
[h] Statens Serum Institute, Copenhagen, Denmark
[i] University of Georgia, Department of Pharmaceutical and Biomedical Sciences, Athens, GA, USA
[j] Uppsala University, Department of Pharmacy, Uppsala, Sweden

Supplementary Methods

Antimicrobial susceptibility testing in urine

The MIC of apramycin in urine was determined by the same standard procedures described in the Methods section, with the exception of substituting cation-adjusted Mueller-Hinton broth with pH-adjusted urine. Morning urine was collected from healthy donors and pooled. Pooled urine was centrifuged and the pH of the supernatant was adjusted to either pH5.0 with 0.1N HCl or to pH7.4 with 0.2 N NaOH, followed by filter sterilization. Results are presented in Table S5.

Supplementary animal efficacy studies

The mouse UTI studies with the MDR pan-aminoglycoside resistant *E. coli* isolate EN0591 (*rmtB*) and *E. coli* isolate EN0355 with limited apramycin susceptibility (MIC of 8 mg/L) were conducted at the Statens Serum Institute in Denmark. Studies were approved by the National Committee of Animal Ethics, Ministry of Environment and food of Denmark. Female C3H/HeJ mice were infected with 50 µL of 10^9 CFU/ml *E. coli* EN591 or *E. coli* EN0355 suspension by intraurethral catheter after treatment with Nurofen Junior (NSAID, 30 mg/kg) for pain alleviation approximately 1 h prior to inoculation. Mice had *ad libido* access to 5% glucose drinking water to induce diuresis from 3 days prior to inoculation onwards. Twice daily, subcutaneous treatment with apramycin (lot.nr: 150710301, Exp. 07.2018) at doses of 0.03-30 mg/kg and vehicle control was started one day post-infection and continued for three consecutive days. At day 1 (baseline) and day 4 post-infection after urine sampling mice were sacrificed by cervical dislocation and bladder and kidneys were removed aseptically for bacterial enumeration. CFU counts were transformed to $\log_{10}$ and plotted against the $\log_{10}$ of administered doses (Fig. S2).

The UTI study with diabetic mice was subcontracted to the University of Texas Medical Branch, studies were approved by the Institutional Animal Care and Use Committee of the University of Texas Medical Branch. Animals were obtained from Charles River laboratories and housed in the animal facilities under standard conditions. *E. coli* ST131 H30Rx strain MVAST072 (M072) was recovered from a chronic cystitis patient. The bacteria were cultured on LB or 5% blood agar at 35-37°C overnight. *E. coli* M072 was passaged to enhance fimbriae and pili formation by static subculture for 15 h at 37°C prior to inoculum preparation. A cohort of one-hundred female C3H/HeN mice at 4-5 weeks of age was used for the study. After acclimation, animals were randomly assigned to one of two groups, a non-diabetic group and a streptozotocin-induced diabetic group. Animals in the diabetic group were subjected to streptozotocin (STZ) diabetic induction (4 daily doses of 65 mg/kg after a 4 h fast). Blood was collected from a nick in the tail and tested with an animal glucometer over the next 14 days to ensure that random blood sugar levels (RBS) increased to diabetic levels (>250). Mice in both groups were infected with 10^7 CFU *E. coli* M072 in 50 µL by the transurethral route on day 0 and twice daily subcutaneous treatment with apramycin (0.1-10 mg/kg) and vehicle control was started 2 days (48 h) post-infection ($n = 10$/ dosing group). Urine was collected daily and animals were sacrificed on day 5 post-infection. The bacterial burden in urine was determined by qPCR after DNA extraction (10 genomes/CFU) and bladder and kidneys were harvested for bacterial enumeration by qPCR as well (Fig. S3).

Supplementary Results

In vitro susceptibility

Table S1 Species distribution in European cUTI cohorts and in the NDARO uropathogenic panel.

	**The Netherlands (1)**		**Poland (2)**		**Switzerland (3)**		**The Netherlands (4)**		**NDARO (Gram-neg.)**
Total cUTI isolates (*n*)	462		237		129		27 922		12 956
Isolates	Species distri-bution (%)	Gentamicin susceptible (%)	Species distri-bution (%)	Amino-glycoside susceptibility	Species distributi on (%)	Gentamicin suceptible (%)	Species distribution (%)	Gentamicin susceptible (%)	Species distribution (*n)*
E. coli	51,3	93,7	65,8	Entero-bacteriaceae: 86% gentamicin susceptible; 93.9% amikacin suceptible	56,2	nd	47,2	94,00	5 049 (39.0%)
K. pneumoniae	9,5	90,9	12,6		4,6	nd	6,7	95,3	5 456 (42.1%)
P. mirabilis	6,7	87,1	8,4		2,3	nd	7,6	91,8	nd
Citrobacter spp.	nd	nd	0,4		3,1	nd	nd	nd	141 (1.1%)
Enterobacter spp.	nd	nd	0,4		0,77	nd	2,1	92,1	488 (3.8%)
P. aeruginosa	5,6	92,3	nd	nd	0,77	nd	5	95	672 (5.2%)
Enterococcus spp.	9,7	nd	nd	nd	9,3	nd	15,1	low level resistance	nd
S. aureus	nd	nd	nd	nd	2,32	nd	2,7	98,7	nd
Streptococci group B	2,6	nd	nd	nd	6,2	nd	2,9	nd	nd

(1) Wijting IEA, Alsma J, Melles DC, Schipper EM, Schuit SCE. Urinary tract infections in a university hospital: pathogens and antibiotic susceptibility. Neth J Med 2019; 77(6): 210-9.

(2) Stefaniuk E, Suchocka U, Bosacka K, Hryniewicz W. Etiology and antibiotic susceptibility of bacterial pathogens responsible for community-acquired urinary tract infections in Poland. *Eur J Clin Microbiol Infect Dis* 2016; **35**(8): 1363-9.

(3) Plate A, Kronenberg A, Risch M, et al. Active surveillance of antibiotic resistance patterns in urinary tract infections in primary care in Switzerland. *Infection* 2019; **47**(6): 1027-35.

(4) Koningstein M, van der Bij AK, de Kraker ME, et al. Recommendations for the empirical treatment of complicated urinary tract infections using surveillance data on antimicrobial resistance in the Netherlands. *PLoS One* 2014; **9**(1): e86634.

Table S2 List of resistance genes applied in determining the genotypic susceptibility of isolates deposited in the NDARO*

	**Apramycin**	**Amikacin**	**Gentamicin**	**Tobramycin**
**Aminoglycoside-modifying enzymes**	*aac(1)-Ia*	*aac(6')-I*	*aac(6')*	*aac(6')*
	aac(3)-IV	*aph(3')-III*	*aac(3)-I*	*aac(3)-II*
	apmA	*aph(3')-VI*	*aac(3)-II*	*aac(3)-III*
		ant(4')	*aac(3)-III*	*aac(3)-IV*
			aac(3)-VII	*aac(2')*
			aph(2'')	*aph(2'')-Ia*
			ant(2'')	*aph(2'')-Id*
			aac(3)-IV	*aph(2'')-Ib*
			apmA	*aph(2'')-II*
				ant(2'')
				ant(4')
				apmA
**Ribosome methyltransferases**	*npmA*	*npmA*	*npmA*	*npmA*
		armA	*armA*	*armA*
		rmtA	*rmtA*	*rmtA*
		rmtB	*rmtB*	*rmtB*
		rmtC	*rmtC*	*rmtC*
		rmtD	*rmtD*	*rmtD*
		rmtF	*rmtF*	*rmtF*
		rmtG	*rmtG*	*rmtG*
		rmtH	*rmtH*	*rmtH*
	kamB	*kamB*	*kamB*	*kamB*

**The minimal determinant of each resistance genotype was identified, meaning that a search for aac(6`) will detect all subtypes of this gene. A search for aac(3)-I implies searching for aac(3)-Ia, b, c etc.*

Table S3 Bacterial and rodent strains used in this study

Species	Strain	Feature	Source
Bacteria			
Escherichia coli	FDA strain Seattle 1946	Reference strain	ATCC 25922
Escherichia coli	J96	UTI model strain	ATCC 700336
Escherichia coli	EN0591	MDR, *rmtB*	University of Zurich
Escherichia coli	EN0335	MIC = MIC_{90} = 8	Uppsala University
Escherichia coli	ST131 H30Rx strain MVAST072 (M072)	MDR, ESBL	University of Texas Medical Branch
Enterobacter cloacae	Various clinical isolates (Table S4)	Uropathogenic, drug resistant	University of Zurich
Klebsiella spp.	Various clinical isolates (Table S4)	Uropathogenic, drug resistant	University of Zurich
Proteus mirabilis	Various clinical isolates (Table S4)	Uropathogenic, drug resistant	University of Zurich
Pseudomonas aeruginosa	Various clinical isolates (Table S4)	Uropathogenic, drug resistant	University of Zurich
Staphylococcus aureus	Various clinical isolates (Table S4)	Uropathogenic, drug resistant	University of Zurich
Rodents			
Mus musculus	C3H-HeJ	UTI model	Jackson Laboratory
Mus musculus	C3H/HeN	Diabetic induction by streptozotocin	Charles River Laboratories
Mus musculus	CD-1	Multipurpose	Envigo
Mus musculus	NMRI	Multipurpose	Envigo
Rattus norwegicus	Sprague-Dawley	Multipurpose	Envigo
Rattus norwegicus	Wistar	Multipurpose	Envigo

Table S4: Minimal inhibitory concentrations determined by broth microdilution antimicrobial susceptibility assays.

SPECIES	STRAIN	GENOTYPE	APR	AMI	TOB	GEN	MER	PIP-TAZ	CTA
E. coli	ATCC 25922	WT	4	2	0.5-1	0.5-1	0.03	2	0.13
E. coli	ATCC 700336	nd	4	2	0.5-1	0.5-1	0.03	2	≤0.06
E. coli	EN0591	RmtB	4	>256	>256	>256	0.06	16	>64
E. coli	EN0335	nd	8	4	1-2	1-2	0.016	2	0.06
E. coli	252 720	ESBL	8	8	32	128	0.03	32	>64
E. coli	254 525	nd	2	4	16	32	≤0.016	64	1-2
E. coli	257 782	ESBL	4	4	32	64	0.03	8	>64
E. coli	260 097	AmpC	2-4	1	0.5	0.25-0.5	0.06	128	>64
E. coli	261 917	ESBL, OXA-48	2-4	1	0.5	0.25-0.5	0.06	>128	>64
E. coli	250 697	ESBL, AmpC	4	2	16	8	0.03	8	>64
E. coli	257 384	ESBL	4	4	32	64	≤0.016	16	>64
E. coli	260 930	nd	8	32	64	128	≤0.016	8	0.5
E. coli	263 311	AmpC	4	4	≤0.25	≤0.25	0.125	128	4-8
E. coli	265 199	ESBL, OXA-48	4	1-2	4	64	0.5	128	>64
E. coli	269 430	ESBL	2-4	4-8	8	0.5	0.03	8	>64
E. coli	259 296	ESBL, NDM-1, RmtB	4	128	256	>256	4	>128	>64
E. coli	263 943	ESBL	4	4	8	16	≤0.016	4	>64
E. coli	264 675	nd	2	1	32	8	≤0.016	64	0.5-1
E. coli	268 263	ESBL	16	32	64	256	0.06	16	>64
E. coli	250 958	ESBL	4	4	32	16	0.06	16	>64
E. coli	252 115	ESBL	4	4	8	32	0.03	16	>64
E. coli	252 467	ESBL, VIM	2-4	8	16	8	0.25-0.5	128	>64
E. coli	259 714	ESBL	8-16	4	1	1	0.125	>128	>64
E. coli	260 063	ESBL	2	1	0.5	0.5	0.06	2	>64
E. cloacae	250 027	cAmpC, ESBL, VIM	1-2	0.5-1	8	8	>16	>128	>64
E. cloacae	259 086	cAmpC, ESBL, VIM	1-2	8	32	16	4	>128	>64
E. cloacae	255 527	cAmpC, OXA-48	1-2	8	16	16	1	>128	>64
E. cloacae	260 779	cAmpC	1-2	8	16	32	0.06	128	>64
E. cloacae	264 713	cAmpC, ESBL	1	1	2	8	0.03	2	>64
K. oxytoca	266 250	ESBL, AmpC	2-4	8	64	256	0.03	>128	32
K. oxytoca	258 351	ESBL	2-4	8	16	64	0.03	32	>64
K. oxytoca	260 643	ESBL	2-4	4	8	64	0.06	32	>64
K. oxytoca	268 204	ESBL	2	1	0.5	0.25-0.5	0.06	>128	8
K. oxytoca	274 763	nd	2	1	≤0.25	0.5	0.06	>128	8
K. pneumoniae	255 915	ESBL	2	1	0.5	0.5	8	>128	>64
K. pneumoniae	263 106	ESBL	2	2	16	32	0.03	128	>64
K. pneumoniae	252 084	ESBL	1	0.5	≤0.25	≤0.25	0.06	8	64
K. pneumoniae	252 695	AmpC	2	2	0.5	0.5	0.06	128	64
K. pneumoniae	268 507	ESBL	2	1-2	4	32	0.03	4	>64
K. pneumoniae	272 212	ESBL	2	0.5	2	32	0.03	1	>64
K. pneumoniae	256 216	ESBL, OXA-48	1-2	4	64	64	>16	>128	>64
K. pneumoniae	251 672	ESBL, RmtB	2-4	>256	>256	>256	0.125	>128	>64
K. pneumoniae	263 115	ESBL	1-2	0.5-1	0.25-0.5	≤0.25	0.25	128	>64
K. pneumoniae	256 706	nd	2	1	≤0.25	≤0.25	0.06	4	0
P. mirabilis	264 373	ESBL	2	1	0.5	0.5	0.03-0.06	0.5	32
P. mirabilis	253 814	AmpC	4-8	2-4	0.5	1	0.06-0.13	0.5	4
P. mirabilis	263 150	NDM-1, RmtB	4	>256	>256	>256	1	16	64
P. mirabilis	255 417	ESBL	256	32	8	8	0.06	0.25	>64
P. mirabilis	270 786	VIM	4	4	4	2	1	128	64
P. mirabilis	254 365	ESBL	8	8	1	1	0.125	0.5	>64
P. mirabilis	266 037	AmpC	8	8	64	128	0.125	64	>64
P. mirabilis	267 794	nd	128	8	32	8	0.06	16	0.5
P. aeruginosa	250 062	MDR	8	32	32	32	16	16	64
P. aeruginosa	265 995	MDR	4-8	2	0.5	2	16	128	>64
P. aeruginosa	257 264	MDR	16	32	64	16	16	8-16	64
P. aeruginosa	257 277	MDR	16-32	16	1	4	2-4	32	>64
P. aeruginosa	257 530	MDR	8	4	0.5	2	16	32	>64
P. aeruginosa	257 892	MDR	16	8	2	8	2-4	2-4	8-16
P. aeruginosa	269 616	MDR	8	64	64	4	>16	32	>64
P. aeruginosa	256 257	MDR	16	32	256	>256	16	128	>64
P. aeruginosa	261 728	MDR	2	2	16	16	16	128	>64

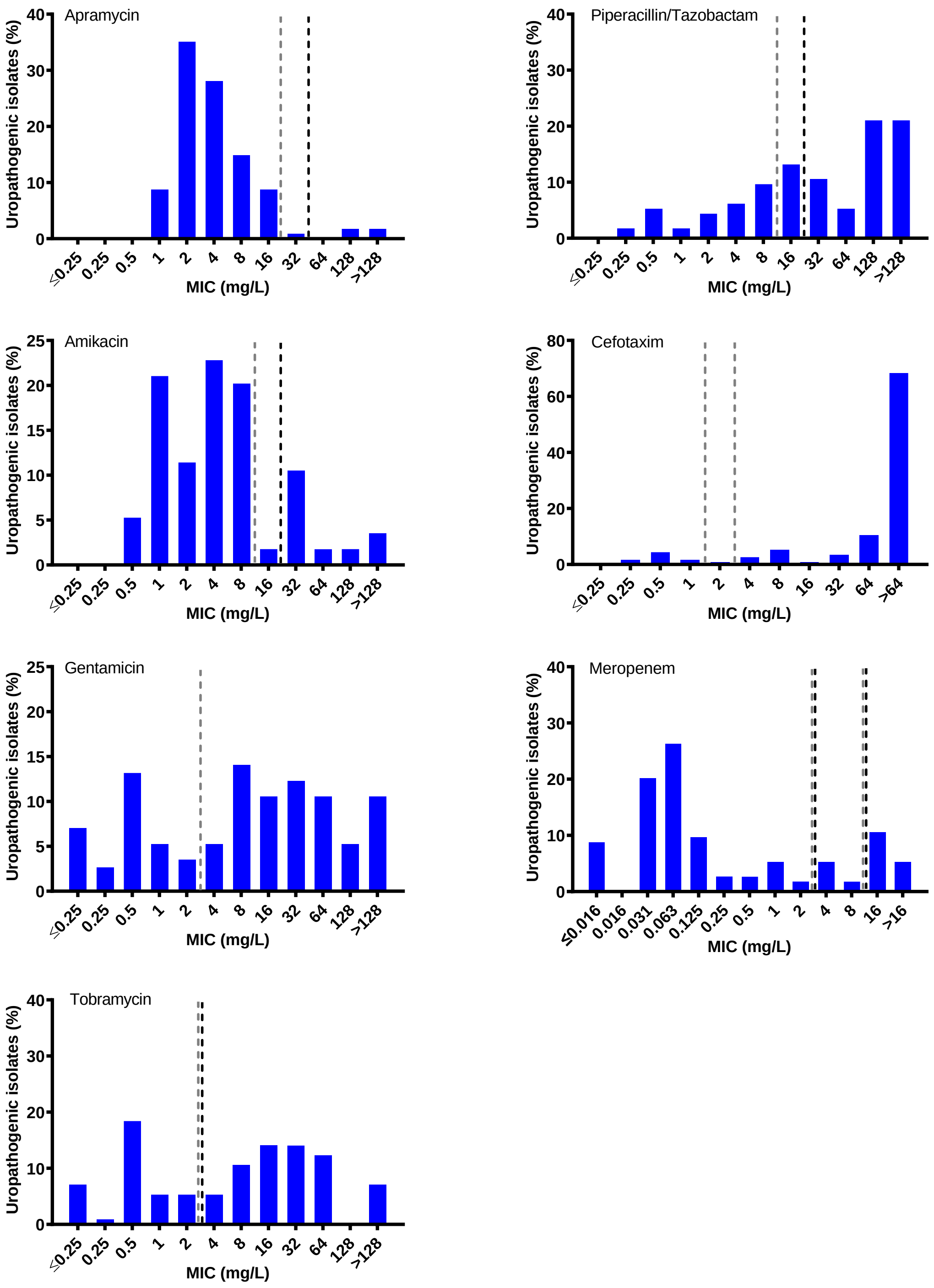

Fig. S1 MIC distribution of apramycin in comparison to other drugs for a panel of uropathogenic bacterial isolates. The MICs of apramycin, amikacin, gentamicin, and tobramycin in comparison to piperacillin/tazobactam, cefotaxime, and meropenem for 57 drug-resistant uropathogenic isolates were determiend by broth microdilution assays according to CLSI standard procedures. The vertical dashed line indicate the EUCAST breakpoints 2021 for Enterobacterales (grey) and *Pseudomonas* spp. (black), respectively. In the case of apramycin, the vertical dashed lines indicate the ECOFF values of 16 and 32 µg/mL, respectively.

In-vivo efficacy studies

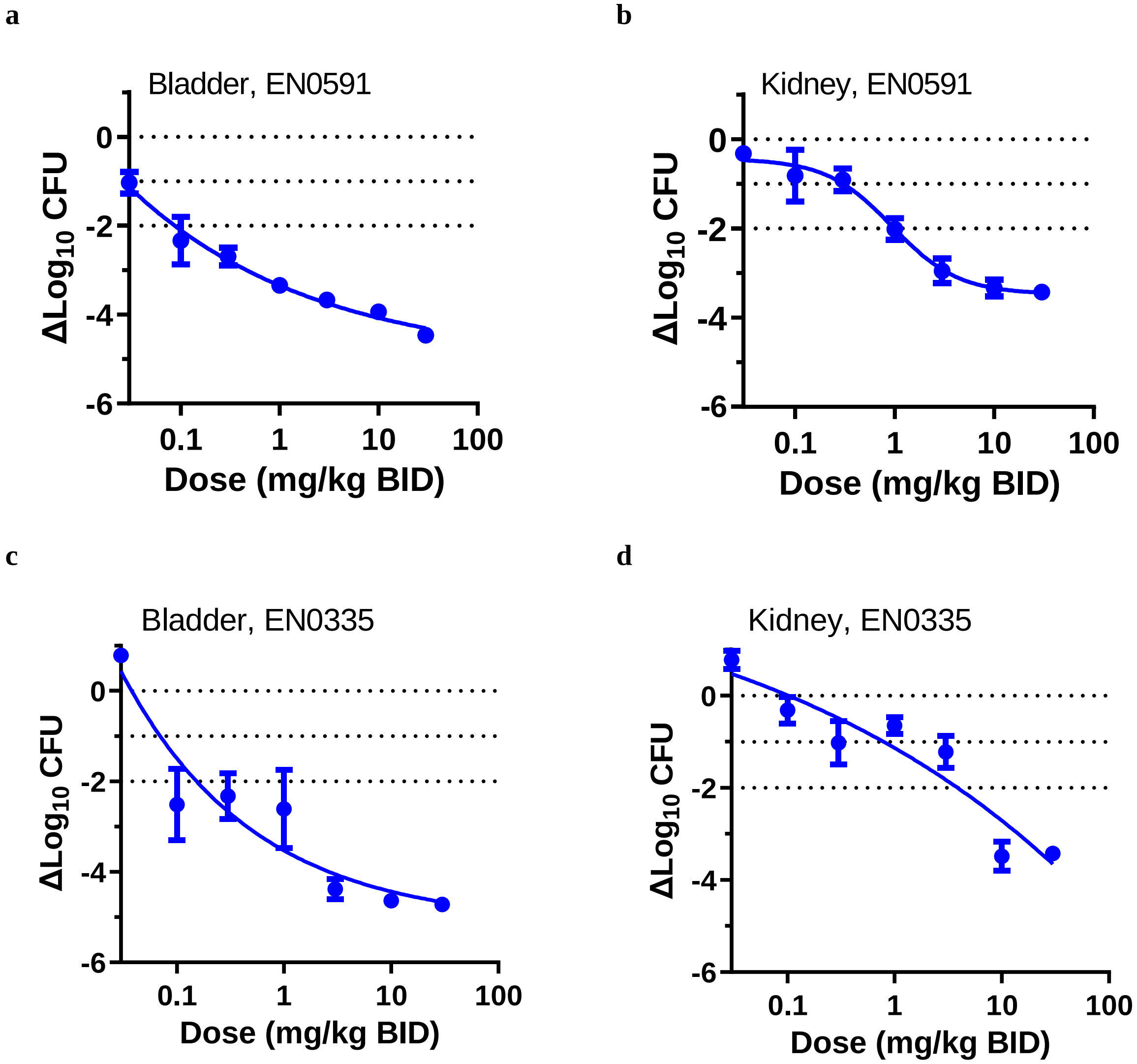

Fig. S2 *In-vivo* efficacy of EBL-1003 in murine infection models with uropathogenic *E. coli* isolates. (**a-b**) CFU count reduction in bladder and kidney of C3H/HeJ mice indicate EBL-1003 efficacy against the pan-aminoglycoside resistant *E. coli rmtB* isolate EN0591 (apramycin MIC of 4 mg/L). Doses of 0.1 and 1 mg/kg and higher significantly reduced mean bacterial load in bladder and kidneys, respectively (study 165-17-05-07; data plotted as mean ± SD, $n = 8$ mice per dose group; ANOVA, Dunnett's multiple comparison test, $P \leq 0.0001$ vs. vehicle controls). (**c-d**) CFU count reduction in kidney and bladder indicate EBL-1003 efficacy against an *E. coli* isolate EN0335 (elevated apramycin MIC of 8 mg/kg). Doses as low as 0.1 and 0.3 mg/kg significantly reduced mean bacterial load in bladder and kidneys, respectively (study 165-17-05-11; data plotted as mean ± SD, $n = 5$ mice per dose group; ANOVA, Dunnett's multiple comparison test, $P = 0.03$ and 0.01, respectively, vs. vehicle controls).

a

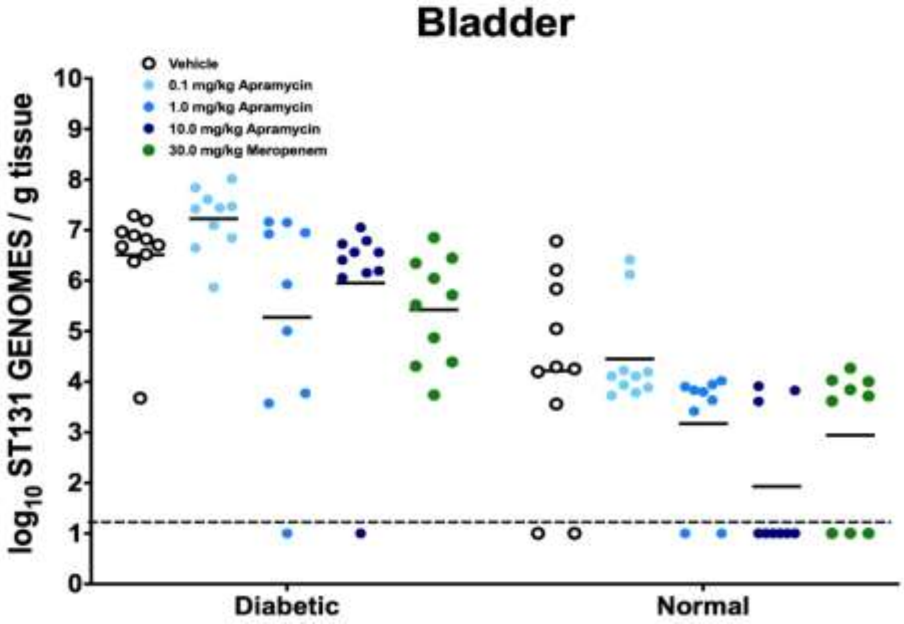

b

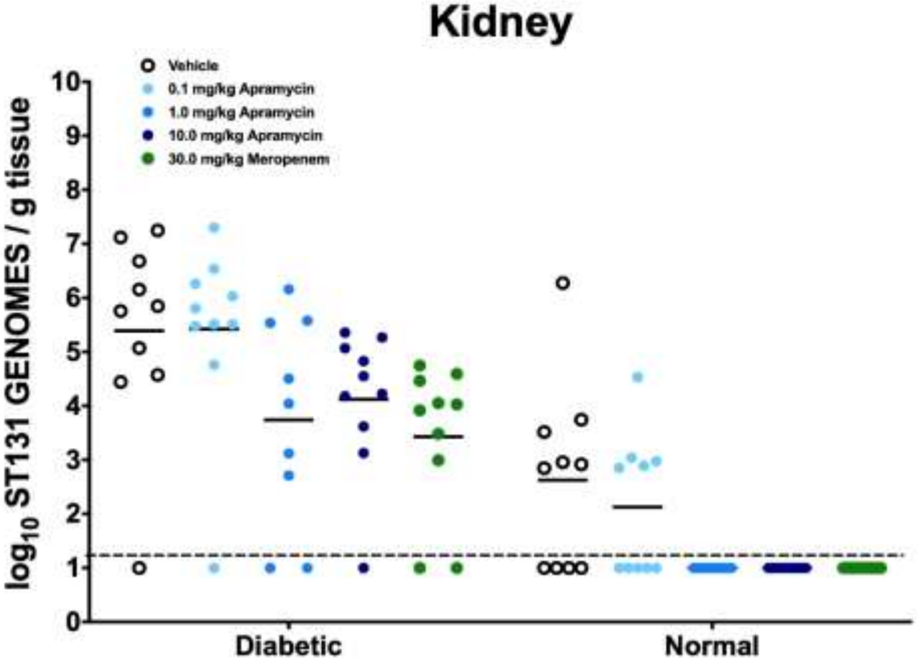

Fig. S3 ***In-vivo*** **potency of apramycin in diabetic murine infection model with the multidrug resistant** ***E. coli*** **M072 isolate.** Subcutaneous injection of 0.1, 1 and 10 mg/kg BID of EBL-1003 (apramycin) or 30 mg/kg of meropenem BID for three days in streptozotocin-induced diabetic or non-diabetic C3H/HeN mice at 4-5 weeks of age after infection with the uropathogenic, multidrug-resistant ESBL *E. coli* ST131 H30Rx strain MVAST072 (M072). The bacterial burden in (**a**) bladder and (**b**) kidney after three days of treatment was assessed by RT-qPCR specific for ST131 genomes (study A02-4).

Table S5: MIC determination for *E. coli* J96 at pH5 and pH7.4 in urine

	MIC (mg/L) in urine		
	**EBL-1003**	**GEN**	**AMI**
**pH7.4**	4	1-2	2-4
**pH5.0**	64-128	32-64	64-128

Table S6: pK_a values by ^{15}N NMR spectroscopy and protonation of individual amino groups

	N-1	N-3	N-2’	N-6’	N-7’	N-3’’	N-4’’	N-4’’’	Net charge	Reference
Apramycin										
pK_a	8.2	6.6	7.7	--	7.5	--	6.7	--		(5)
pK_a	8.1	6.6	7.6	--	7.4	--	6.6	--		(6)
HA^+										
pH7.4	83%	14%	61%	--	50%	--	14%	--	+2.22	
pH6.5	98%	56%	93%	--	89%	--	56%	--	+3.90	
pH6.0	99%	80%	98%	--	96%	--	80%	--	+4.53	
pH5.0	100%	98%	100%	--	100%	--	98%	--	+4.94	
Gentamicin										
pK_a	8.9	7.2	8.2	10.0	--	9.3	--	--		(7)
HA^+										
pH7.4	97%	39%	86%	100%	--	99%	--	--	+4.20	
pH6.5	100%	83%	98%	100%	--	100%	--	--	+4.81	
pH6.0	100%	94%	99%	100%	--	100%	--	--	+4.93	
pH5.0	100%	99%	100%	100%	--	100%	--	--	+4.99	
Amikacin										
pK_a	>10	7.6	--	8.9	--	8.1	--	9.7		(8)
pK_a	>10	7.6	--	8.8	--	8.1	--	9.9		(9)
HA^+										
pH7.4	100%	61%	--	96%	--	83%	--	100%	+4.41	
pH6.5	100%	93%	--	100%	--	98%	--	100%	+4.90	
pH6.0	100%	98%	--	100%	--	99%	--	100%	+4.97	
pH5.0	100%	100%	--	100%	--	100%	--	100%	+5.00	

(5) Dorman DE, Paschal JW, and Merkel KE (1976) J. Am. Chem. Soc. 98:6885-6888. DOI: 10.1021/ja00438a020
(6) Paschal JW and Dorman DE (1978) Organic Magnetic Resonance. 11:632-634. DOI: 10.1002/mrc.1270111210
(7) Lesniak W, Mc Laren J, Harris WR, Pecoraro VL, and Schacht J. (2003) Carbohydr Res. 338:2853-62. DOI: 10.1016/j.carres.2003.08.005
(8) Cox JR and Serpersu EH (1997) Biochemistry 36:2353–2359. DOI: 10.1021/bi9626822
(9) Alkhzem AH, Woodman TJ, and Blagbrough IS (2020) ACS Omega 5:21094-21103. DOI: 10.1021/acsomega.0c02744

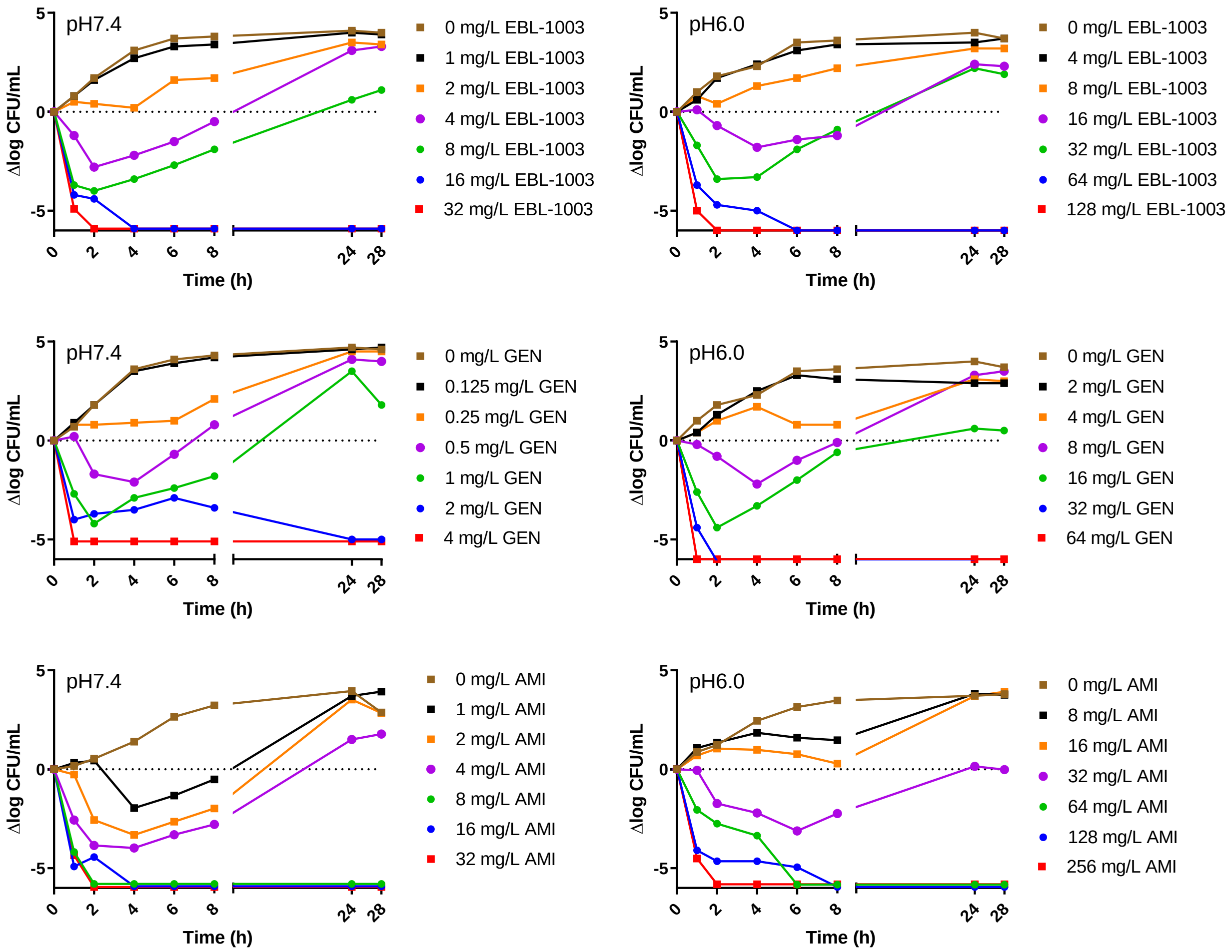

Fig. S4 Time-kill analysis of EBL-1003 (apramycin), in comparison to gentamicin (GEN) and amikacin (AMI) with *E coli* strain J96 at neutral and acidic pH.

Pharmacokinetics and nephrotoxicity

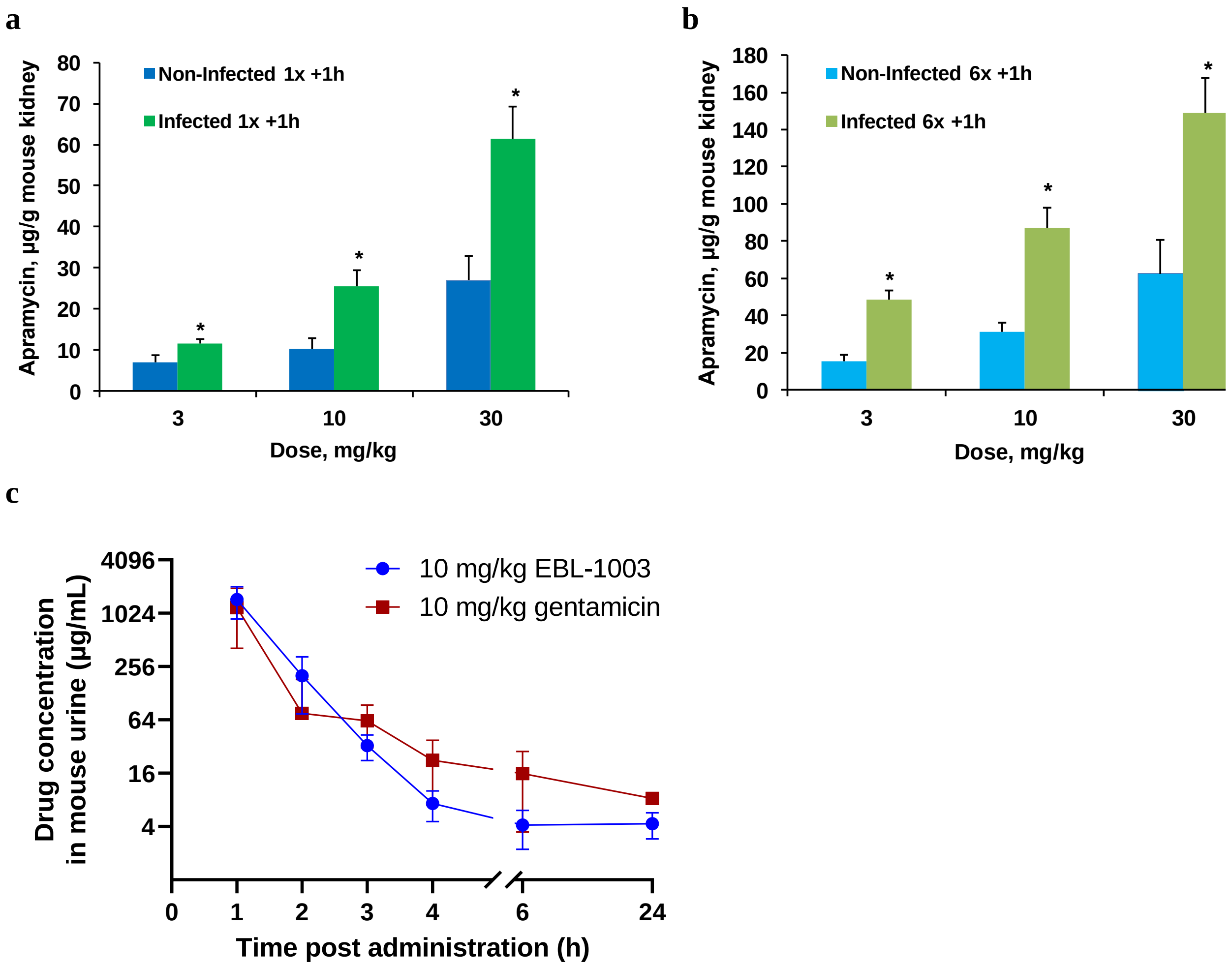

Fig. S5 Apramycin exposure in non-infected and infected murine kidney tissue and in urine after subcutaneous administration of EBL-1003. (**a**) Apramycin concentration in kidneys one hour after a single dose of EBL-1003 in healthy and infected mice. Healthy NMRI mice (study EXP-18-BN9099) and C3H/HeJ mice infected by intraurethral inoculation of 5×10^7 CFU of *E. coli* isolate EN591 one day prior to start of treatment (study 165-17-05-07 and EXP-18-BN9096) were treated subcutaneously with 3, 10, or 30 mg/kg BID of EBL-1003 (data plotted as mean±SD of $n = 4$ healthy animals or $n = 8$ infected animals per dose group). (**b**) Apramycin concentration in the kidneys of healthy and infected animals after three days of repeated BID dosing (total of six doses). Kidneys were harvested one hour after administration of the final dose. Apramycin concentrations in the kidneys of infected mice were compared to those in non-infected mice using Student's t-tests. $P < 0.05$ was considered statistically significant (*). (**c**) Apramycin concentration in mouse urine in comparison to gentamicin after a single subcutaneous dose of 10 mg/kg (data plotted as mean of $n = 4$ animals per time point; study EXP-17-BN9069).

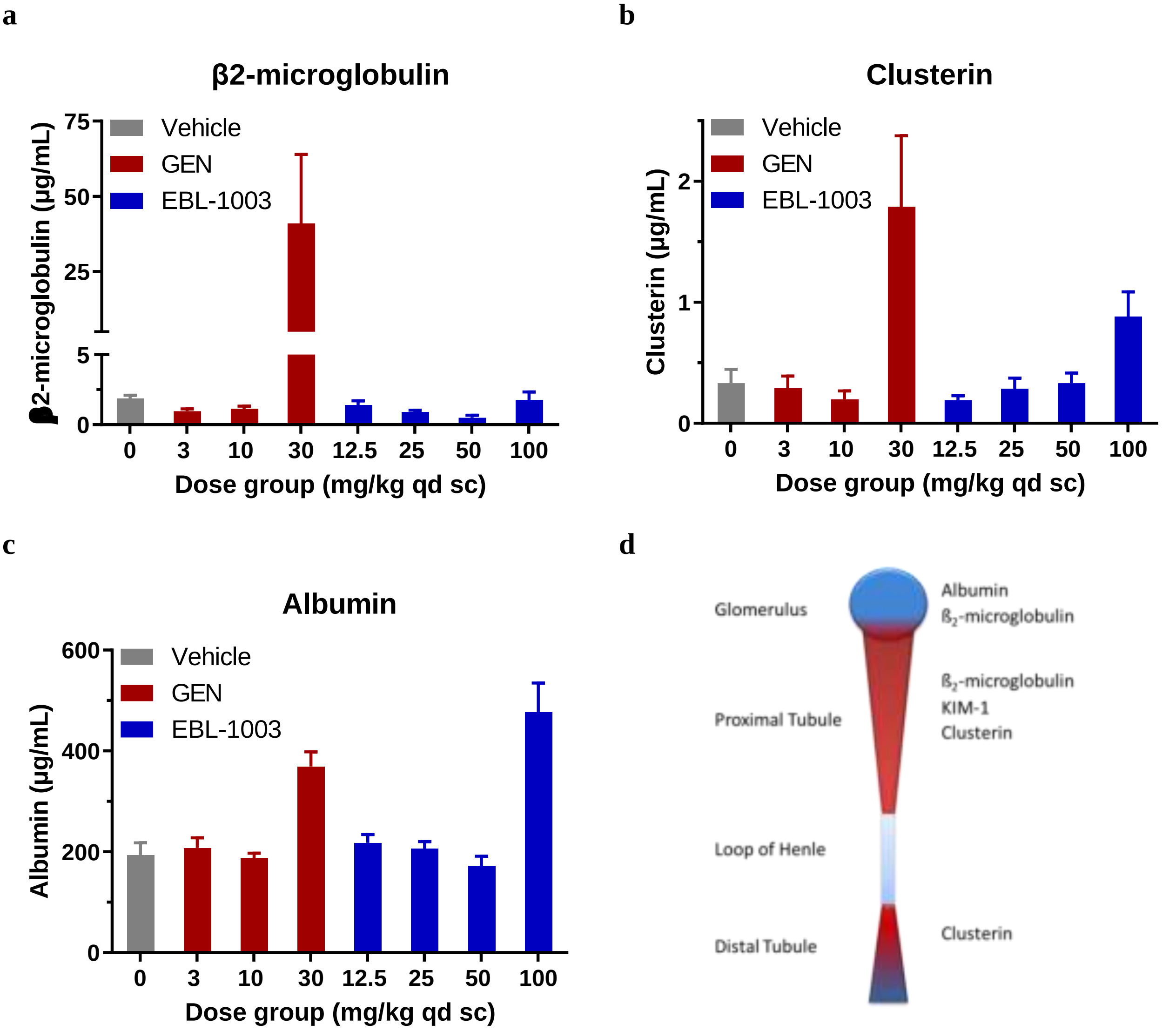

Fig. S6 Quantification of nephrotoxic biomarkers in adult rats treated with gentamicin or apramycin. (**a**) β2-microglobulin concentration in the urine of adult Sprague-Dawley rats (≥14 weeks old at first dose) after 14 days of once-daily subcutaneous treatment with gentamicin (GEN) or apramycin (EBL-1003), respectively. (**b**) Clusterin, and (**c**) albumin concentrations in the same urine samples. All values are plotted as mean±SEM (*n* = 5 animals per dose group; study EXP-17-BM8077). (**d**) Renal schematic indicating the likely site of cellular damage based on the biomarker profile. Aminoglycoside toxicity is known for primarily targeting the proximal tubular cells.

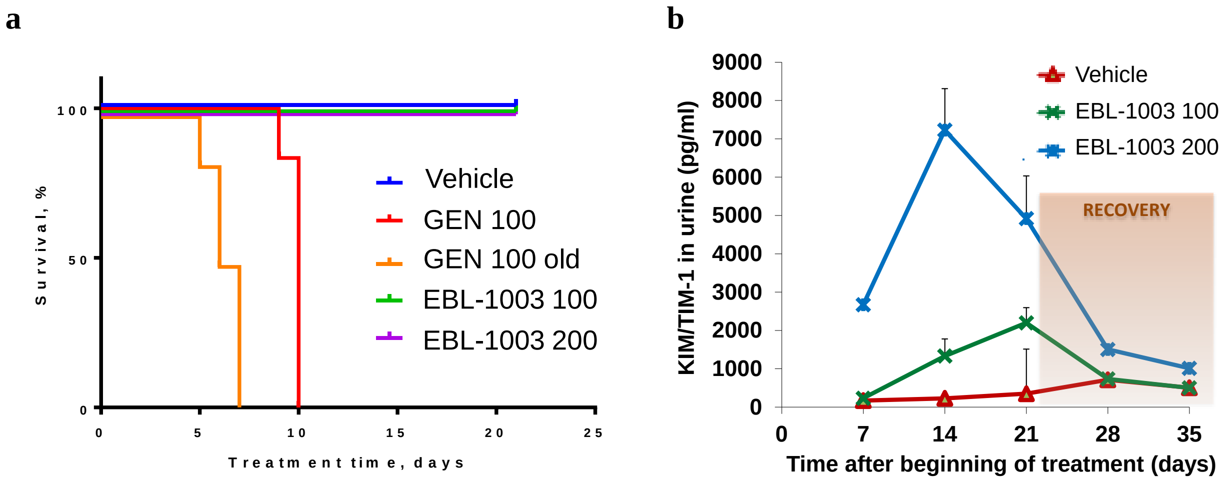

Fig. S7 Survival and recovery of rats dosed with 100 and 200 mg/kg of apramycin (EBL-1003) or gentamicin (GEN) for up to 21 days. (**a**) Survival curves of rats treated with 100 mg/kg q.d. of gentamicin (GEN), 100 or 200 mg/kg q.d. of apramycin (EBL-1003), or vehicle control (physiologic saline) for 21 days (*n* = 5 animals per dose group). For the gentamicin dosing group, we included a control group that was 18 weeks old at start of treatment (GEN 100 old) to determine a possible effect of age on nephrotoxic vulnerability (study EXP-17-BM8056). (**b**) Renal recovery of rats after 21 days of treatment with 100 or 200 mg/kg of EBL-1003 was monitored for another two weeks after the last dose (35 days after start of treatment; study EXP-17-BM8056).

Supplementary References

1. Wijting IEA, Alsma J, Melles DC, Schipper EM, Schuit SCE. Urinary tract infections in a university hospital: pathogens and antibiotic susceptibility. Neth J Med. 2019;77(6):210-9.

2. Stefaniuk E, Suchocka U, Bosacka K, Hryniewicz W. Etiology and antibiotic susceptibility of bacterial pathogens responsible for community-acquired urinary tract infections in Poland. Eur J Clin Microbiol Infect Dis. 2016;35(8):1363-9.

3. Plate A, Kronenberg A, Risch M, Mueller Y, Di Gangi S, Rosemann T, et al. Active surveillance of antibiotic resistance patterns in urinary tract infections in primary care in Switzerland. Infection. 2019;47(6):1027-35.

4. Koningstein M, van der Bij AK, de Kraker ME, Monen JC, Muilwijk J, de Greeff SC, et al. Recommendations for the empirical treatment of complicated urinary tract infections using surveillance data on antimicrobial resistance in the Netherlands. PLoS One. 2014;9(1):e86634.

5. Dorman DE, Paschal JW, Merkel KE. 15N nuclear magnetic resonance spectroscopy. The nebramycin aminoglycosides. J Am Chem Soc. 1976;98(22):6885-8.

6. Paschal JW, Dorman DE. Determination of pKa values using 15N and 13C nuclear magnetic resonance spectroscopy. The case of apramycin. Organic Magnetic Resonance. 1978;11(12):632-4.

7. Lesniak W, Mc Laren J, Harris WR, Pecoraro VL, Schacht J. An isocratic separation of underivatized gentamicin components, 1H NMR assignment and protonation pattern. Carbohydr Res. 2003;338(24):2853-62.

8. Cox JR, Serpersu EH. Biologically important conformations of aminoglycoside antibiotics bound to an aminoglycoside 3'-phosphotransferase as determined by transferred nuclear Overhauser effect spectroscopy. Biochemistry. 1997;36(9):2353-9.

9. Alkhzem AH, Woodman TJ, Blagbrough IS. Individual pK a Values of Tobramycin, Kanamycin B, Amikacin, Sisomicin, and Netilmicin Determined by Multinuclear NMR Spectroscopy. ACS Omega. 2020;5(33):21094-103.
